# Supplementary material for: Selective branch formation in ethylene polymerization to access precise ethylene-propylene copolymers
Source: Nat Commun. 2022 Feb 7;13:725. doi: 10.1038/s41467-022-28282-z (PMC8821618; doi:10.1038/s41467-022-28282-z)
Supplement: Supplementary file 1 — Supplementary Information [file 41467_2022_28282_MOESM1_ESM.pdf]

Supplementary Information

Selective branch formation in ethylene polymerization to access precise ethylene-propylene copolymers

Yuxing Zhang,[1,2] Xiaohui Kang[3,*] & Zhongbao Jian[1,2,*]

[1] State Key Laboratory of Polymer Physics and Chemistry, Changchun Institute of Applied Chemistry, Chinese Academy of Sciences, Renmin Street 5625, Changchun 130022, China
[2] University of Science and Technology of China, Hefei 230026, China
[3] College of Pharmacy, Dalian Medical University, Dalian, 116044 China

Contents

Supplementary Notes	1
Supplementary Methods	4
Supplementary Tables	7
Supplementary Figures	8
Supplementary References	36

Supplementary Notes

1. Computational Details

In present work, all quantum chemical computations were performed by using the Gaussian16 package of programs.[1] Each optimized structure was optimized at GGA BP86[2–4]/BSI level, and was subsequently characterized as a minimum (N_{imag} = 0) or a transition state (N_{imag} = 1) by harmonic vibration frequencies which providing thermodynamic data. The transition state structures are shown to connect the reactant and product on either side via intrinsic reaction coordinate (IRC) following. In the BSI, the C and H atoms in gray part (Supplementary Figure 1) were considered by 3-21G basis set, the other C, H, O, and N atoms were calculated by the 6-31G* basis set, and the Ni and Pd atoms were treated by the quasi-relativistic LANL2DZ ECP effective core potential.[5] To obtain more reliable relative energies, the single-point calculations of optimized structures were carried out at the level of BP86-D3 (BP86 with Grimme's DFT-D3 correction)[6,7]/BSII, taking into account solvation effect of toluene with the SMD[8] solvation model. In BSII, the 6-311G(d, p) basis set was used for C, H, O, and N atoms and the Ni and Pd atoms were treated by the Stuttgart/Dresden effective core potential (ECP) and the associated basis sets.[9] Therefore, unless otherwise mentioned, the free energy (ΔG, 298.15 K, 1 atm) in solution, which was used for description of energy profiles, was obtained from the solvation single-point calculation and the gas-phase Gibbs free energy correction.[10]

In energy decomposition analysis (EDA), the energies of two fragments, viz., the monomer moiety (A) and the remaining metal complex (B), in the TS geometries were evaluated via single-point calculations. Such single-point energies of the fragments and the energy of TS were used to estimate the interaction energy ΔE_{int}. These energies, together with the energies of the respective fragments in their optimal geometry, allow for the estimation of the deformation energies of the two fragments, ΔE_{def}(A) and ΔE_{def}(B). As the energy of the TS, ΔE_{TS}, is evaluated with respect to the energy of the two separated fragments, the relation $\Delta E_{TS} = \Delta E_{int} + \Delta E_{def}(A) + \Delta E_{def}(B)$ holds.

2. Detailed Discussion on Mechanisms of Nickel and Palladium System

Nickel system. The intriguing branching character stimulated us to study the related mechanism of ethylene polymerization by nickel species **ipty-Ni** and palladium species **ipty-Pd** through using DFT calculations (Supplementary Figures 2 and 3). At first, the reaction starts from the β-H elimination (BHE) based on a β-agostic species $\mathbf{1_{\beta\text{-}T}}$ to give a propylene-coordinated complex **1**. This process, which overcomes a free energy barrier ($\mathbf{TS_{1BHE}}$) of 12.9 kcal mol^{-1}, is endergonic by 9.5 kcal mol^{-1}. This unstable **1** goes through a slightly lower $\mathbf{TS_{12reins}}$ with an energy barrier of 12.0 kcal mol^{-1} to complete propylene re-insertion with a 2,1-manner, generating a new β-agostic intermediate **2**. By comparison, **2** is the more both kinetically and thermodynamically favorable than $\mathbf{1_{\beta\text{-}T}}$. Therefore, a new ethylene coordination separating β-agostic-H based on **2** is considered, and is found to be feasible via an energy barrier of 12.7 kcal mol^{-1}, leading to **3**. Finally, an ethylene insertion into the Ni–isopropyl bond of **3** occurs via a $\mathbf{TS_{34ins}}$ with an energy barrier of 10.0 kcal mol^{-1} to generate a methyl-branching **4**, accompanied with an energy release of 15.1 kcal mol^{-1}.

Starting from **4**, two possible pathways, viz., the direct ethylene coordination–insertion ($\mathbf{4 \rightarrow TS_{45coor} \rightarrow 5 \rightarrow TS_{56ins} \rightarrow 6}$) and the BHE–2,1-reinsertion ($\mathbf{4 \rightarrow TS_{5BHE} \rightarrow 5_{BHE\text{-}C} \rightarrow TS_{56reins} \rightarrow 6_{reins}}$), were calculated. The former pathway has a total energy barrier of 14.2 ($\Delta G_1^{\ddagger}$, -3.4–(-17.6), $\mathbf{TS_{45coor}}$) kcal mol^{-1} and is exergonic by 11.4 (-29.0–(-17.6)) kcal mol^{-1}. By contrast, a higher energy barrier of 18.1 ($\Delta G_2^{\ddagger}$, 0.5–(-17.6), $\mathbf{TS_{56reins}}$) kcal mol^{-1} and an endergonic character (5.0 = -12.6–(-17.6), $\mathbf{6_{reins}}$) in the latter case suggest that 1,2-Me_2 unit ($\mathbf{6_{reins}}$) is inaccessible in both kinetics and thermodynamics,

which is in line with the experimental observation. To gain more insight on above difference, the geometries of **TS$_{45coor}$** and **TS$_{56reins}$** are carefully compared as shown in Supplementary Figure 4. In comparison with **1$_{β\text{-}T}$** and **TS$_{45coor}$**, 2,1-reinsertion of 3-methyl-1-butene (MB) in **TS$_{56reins}$** results in the bigger repulsion to phenyls of the auxiliary ligand as suggested by the bigger dihedral angles (119–125° vs 118°–119° in **1$_{β\text{-}T}$** and 118–122° in **TS$_{45coor}$**). In addition, the C=C bond of MB deviates from the plane of the Ni-involved ring, which could make MB insertion with a 2,1-mode into Ni–H bond more difficult. Subsequent ethylene insertion into **6$_{reins}$** (**6$_{reins}$→TS'$_{67coor}$→7'→TS'$_{78ins}$→8'**) has a higher energy barrier of 21.9 ($ΔG_3^‡$, 4.3–(-17.6), **TS'$_{67coor}$**) kcal mol^{-1}, further confirming that the reverse reaction of 1,2-Me_2 unit formation more easily occurs.

To further theoretically access the origin of selectivity on exclusive methyl branches, that is why 1,4-Me_2 unit can be produced but longer-chain branch units such as 1,3-Me/Et unit are unavailable, we also investigated further ethylene insertion and BHE based on **6**. As indicated in Supplementary Figure 2, the direct ethylene coordination-insertion overcomes a total energy barrier of 13.1 ($ΔG_4^‡$, -15.9–(-29.0), **TS$_{67coor}$**) kcal mol^{-1} and releases an energy of 12.4 (-41.4–(-29.0)) kcal mol^{-1}, yielding a precursor (**8**) of 1,x-Me_2 units (x = 2n+4, n = 1, 2, 3...). To obtain 1,4-Me_2 unit and 1,3-Me/Et unit, a BHE and 2,1-reinsertion from **6** was calculated. Notably, locating 5-methyl-1-hexene-coordinated intermediate **7$_{BHE\text{-}C}$** is fruitless, and just one transition state **TS$_{78reins}$** with an energy barrier of 13.9 ($ΔG_5^‡$, -15.1–(-29.0)) kcal mol^{-1} is obtained between **6** and **8$_{reins}$** (1,4-Me_2 unit, observed in the experiment). And the relaxed scans (Supplementary Figures 5 and 6) on C2–H1 distance and dihedral angle ∠H1-Ni-C2-C1 from **TS$_{78reins}$** to **6** direction indicate that neither a maximum nor a minimum point is found, thus further confirming our results. Then, **8$_{reins}$** isomerizes to a more stable **8$_{reins\text{-}iso}$** (also 1,4-Me_2 unit). It is noteworthy that the generations of 1,6-Me_2 unit precursor ($ΔG_4^‡$ = 13.1 kcal mol^{-1}) and 1,4-Me_2 unit ($ΔG_5^‡$ = 13.9 kcal mol^{-1}) indicate the similar energy barriers, suggesting that they both can be obtained. This shows a good agreement with experimental results.

Furthermore, in following BHE and reinsertion from **8$_{reins\text{-}iso}$** to **9$_{reins}$** (1,3-Me/Et unit), we cannot also locate 5-methyl-2-hexene (MH)-coordinated intermediate **8$_{BHE\text{-}C}$** and just obtain one transition state **TS$_{8BHE}$**. It is also further confirmed by the relaxed scan (Supplementary Figure 7) on the dihedral angle ∠H1-Ni-C2-C1 from **8$_{reins\text{-}iso}$** to **9$_{reins}$**. Unlike 1,4-Me_2 unit, the formation of 1,3-Me/Et unit needs going through a higher **TS$_{8BHE}$** with an energy barrier of 24.3 ($ΔG_8^‡$, -9.0–(-33.3)) kcal mol^{-1} and is an apparently unfavorable process, which is consistent with experimental findings. To have a better understanding on above differences, the optimized geometrics for **TS$_{78ins}$**, **TS$_{78reins}$**, and **TS$_{8BHE}$** were comparatively analyzed as shown in (Supplementary Figure 8). In **TS$_{8BHE}$**, the bigger steric repulsion between MH and phenyl groups, as suggested by the bigger dihedral angles (126/129° *vs.* 122/121° in **TS$_{78ins}$** and 121/120° in **TS$_{78reins}$**), can account for the less stability of **TS$_{8BHE}$** in comparison with **TS$_{78ins}$** and **TS$_{78reins}$**.

Beyond those, the possibilities of further polymerization based on **8$_{reins}$** and **8$_{reins\text{-}iso}$** were also considered. It was found that the ethylene coordination-insertion (**8$_{reins}$→TS$_{89coor}$→9→TS$_{910ins}$→10**) into **8$_{reins}$** owns an energy barrier of 10.3 ($ΔG_6^‡$, -19.4–(-29.7)) kcal mol^{-1} and is exergonic by 14.5 ((-44.2–(-29.7))) kcal mol^{-1}, while the process (**8$_{reins\text{-}iso}$→TS'$_{89coor}$→9'→TS'$_{910ins}$→10**) involving **8$_{reins\text{-}iso}$** overcomes a higher energy barrier of 24.2 ($ΔG_7^‡$, -9.1–(-33.3)) kcal mol^{-1} and is a less exergonic process (-7.9 = -41.2–(-33.3) kcal mol^{-1}). Therefore, the subsequent insertion on basis of **8$_{reins}$** is smooth. Further energy decomposition (Supplementary Figure 9) indicate that the less stability of **TS'$_{910ins}$** can be ascribed to the bigger repulsion of poly(ethylene) chain/ethylene monomer and auxiliary ligand, as suggested by a bigger deformation energy ($ΔE_{def} = ΔE_{def(A)} + ΔE_{def(B)}$ = 77.3 *vs.* 55.5 kcal mol^{-1} in **TS$_{910ins}$**), the bigger dihedral angles ($∠α_1β_1γ_1/∠α_1β_2γ_2$: 124/123° in **TS'$_{910ins}$** *vs.* 121/122° in **TS$_{910ins}$**) and a farther distance($γ_1–γ_2$ = 8.24 Å in **TS'$_{910ins}$** *vs.* 7.96 Å in **TS$_{910ins}$**) between two phenyl centers of ligand. Aforementioned findings allow us to conclude that the unique ancillary ligand forms a suitable channel for an exclusive formation of small methyl branches and a precise

distribution of 1,2n+4-Me_2 units (n = 0, 1, 2, 3...) in highly branched polyethylenes.

Palladium system. Since **ipty-Pd** also generated highly branched polyethylenes with exclusive methyl branches and precise branch distribution, the same reaction pathways as those of **ipty-Ni** were also calculated in palladium mediated ethylene polymerization to investigate the discrepancy of between nickel and palladium species (Supplementary Figure 3). The results indicate that the relative energies of all Pd-involved π-complexes (**1**, **3**, **5**, $\mathbf{5_{BHE\text{-}C}}$, **7**, **7'**, **9**, and **9'**) are apparently lower than those in the nickel system. Moreover, the geometric characters of transition states ($\mathbf{TS_{1BHE}}$, $\mathbf{TS_{12reins}}$, $\mathbf{TS_{5BHE}}$, $\mathbf{TS_{56reins}}$, $\mathbf{TS_{7BHE}}$, $\mathbf{TS_{78reins}}$, and $\mathbf{TS_{8BHE}}$, Supplementary Figure 10) involved in the process of branch formation are similar to π mode complexes, thus showing lower relative energies than those of the nickel case. Therefore, the Pd with a bigger ionic radius (0.86 Å) shows a stronger preference for stabilizing π-mode complexes in comparison with the Ni (0.69 Å) counterparts, which is consistent with previous reports. As aforementioned, the formation of 1,2-Me_2 unit ($\mathbf{6_{reins}}$) in the Pd system just overcomes an energy barrier of 9.7 ($\Delta G_2^{‡}$, -6.6–(-16.3)) kcal mol^{-1} and is endergonic by 2.3 (-14.0–(-16.3)) kcal mol^{-1}. Based on this, further ethylene insertion needs to surmount a $\mathbf{TS'_{67coor}}$ via a higher energy barrier of 17.8 ($\Delta G_3^{‡}$, 1.5–(-16.3)) kcal mol^{-1} than that ($\Delta G_1^{‡}$, 15.5 kcal mol^{-1}) of direct ethylene coordination-insertion into **4**. Hence, the thermodynamic instability ($\mathbf{6_{reins}}$: -14.0 *vs.* **4**: -16.3) and the more difficult further insertion of ethylene make it hard for producing 1,2-Me_2 unit. Likewise, on the basis of **6**, the BHE-reinsertion ($\mathbf{6 \rightarrow TS_{7BHE} \rightarrow 7_{BHE\text{-}C} \rightarrow TS_{78reins} \rightarrow 8_{reins}}$) leading to 1,4-$Me_2$ unit just has a free energy barrier of 7.3 ($\Delta G_5^{‡}$, -20.7–(-28.0)) kcal mol^{-1} and subsequent ethylene coordination-insertion from $\mathbf{8_{reins}}$ is accessible (with an energy barrier of $\Delta G_6^{‡}$ = 15.0 kcal mol^{-1} *vs*. $\Delta G_7^{‡}$ = 27.3 kcal mol^{-1} based on $\mathbf{8_{reins\text{-}iso}}$). Beyond that, $\mathbf{8_{reins\text{-}iso}}$ comes across a 17.2 kcal mol^{-1} ($\Delta G_8^{‡}$) energy barrier and is endergonic by 3.5 kcal mol^{-1} to produce $\mathbf{9_{reins}}$ (1,3-Me/Et unit). These results imply the formation of 1,4-Me_2 unit via an easy isomerization (chain walking, low 7.3 kcal mol^{-1}) and a comparable barrier (15.0 kcal mol^{-1} to compound **10** vs 15.5 kcal mol^{-1} to compound **8**) of subsequent ethylene insertion. The slightly unfavorable kinetics (+2.2 kcal mol^{-1}) indicates an impossibility of the formation of 1,3-Me/Et unit at low temperature but a possibility at higher temperature. This agrees the presence of longer branches in palladium system at elevated temperatures.

Supplementary Methods

General Procedures: All syntheses involving air- and moisture sensitive compounds were carried out using standard Schlenk-type glassware (or in a glove box) under an atmosphere of nitrogen. All solvents were purified from the MBraun SPS system. NMR spectra for the ligand, complex, and polymers were recorded on a Bruker AV400 (^{1}H: 400 MHz, ^{13}C: 100 MHz) or a Bruker AV500 (^{1}H: 500 MHz, ^{13}C: 125 MHz). NMR assignments were confirmed by ^{1}H−^{1}H COSY experiments when necessary. The molecular weights (M_n) and molecular weight distributions (M_w/M_n) of polyethylenes and copolymers were measured by means of gel permeation chromatography (GPC) on a PL-GPC 220-type high-temperature chromatograph equipped with three PL-gel 10 μm Mixed-B LS type columns at 150 ℃. Melting temperature (T_m) of copolymers were measured through DSC analyses, which were carried out on a Mettler TOPEM TM DSC Instruments under nitrogen atmosphere at heating and cooling rates of 10 ℃/min (temperature range: -100−160 ℃). Elemental analysis were performed at the National Analytical Research Centre of Changchun Institute of Applied Chemistry.

X-Ray diffraction: Data collections were performed at −100 ℃ on a Bruker SMART APEX diffractometer with a CCD area detector, using graphite-monochromated Cu Kα radiation (λ = 1.54178 Å). The determination of crystal class and unit cell parameters was carried out by the SMART program package.[11] The raw frame data were processed using SAINT and SADABS to yield the reflection data file.[12] All structures were solved and refined by full-matrix least-squares procedures on F^2 using SHELXTL or Olex2.[13] Refinement was performed on F^2 anisotropically for all non-hydrogen atoms by the full-matrix least-squares method. The hydrogen atoms were placed at the calculated positions and were included in the structure calculation without further refinement of the parameters.

Exceptions and special features: For **ipty-Ni**, the program SQUEEZE[14] was used to remove mathematically the effect of the solvent. The quoted formula and derived parameters are not included the squeezed solvent molecules.

Explanations of B level alerts on ipty-Ni single crystal structure: The “THETM01 ALERT 3 B” and “PLAT413 ALERT 2 B” alerts are generated due to that the quality of this single crystal is not very good and there is a large amount of H atom disorder in the structure. We tried to recollect the data from a crystal of higher quality, but attempts to obtain good crystals by using various available solvent systems were failed. The used data was collected from the best crystal that we obtained. We ensured that the two alerts do not affect the structure and the analyzed data was correct.

Materials: α-diimine ligand was prepared using the literature procedure.[15]

Preparation of Catalyst

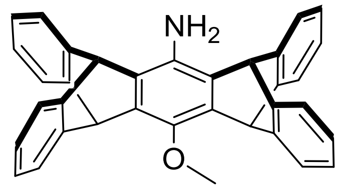

Preparation of pentiptycene aminoanisole: To a solution of pentiptycene aminophenol (3 g, 6.5 mmol) in 100 mL dry DMF at r.t. was added NaH (468 mg, 19.5 mmol) under nitrogen. The mixture foamed and was warmed. CH_3I (0.6 mL, 9.75 mmol) was added after the bubbling ceased and the mixture was stirred at r.t. for 24 h. The reaction was quenched by adding 400 mL of distilled water and the aqueous layer was extracted with 3 × 50 mL of CH_2Cl_2. The combined organic phases were washed with 3 ×50 mL of distilled water and 50 mL of brine. Then the organic phase was separated and dried with sodium sulfate. After evaporation of the solvent the pure product was obtained as white solid (2.6 g, 84.1% yield). This method was similar to our previous study.[15]

^{1}H NMR (500 MHz, 298 K, $CDCl_3$, 7.26 ppm): δ =7.27-7.36 (m, 8H, aryl-*H*), 6.98-6.88 (m, 8H, aryl-*H*), 5.67 (s, 2H, C*H*Ph_2), 5.39 (s, 2H, C*H*Ph_2), 3.85 (s, 3H, OCH_3) ppm.

Elemental analysis: Anal. Calcd for $C_{35}H_{25}NO$: C, 88.39; H, 5.30; N, 2.95. Found: C, 88.16; H, 5.27; N, 2.90.

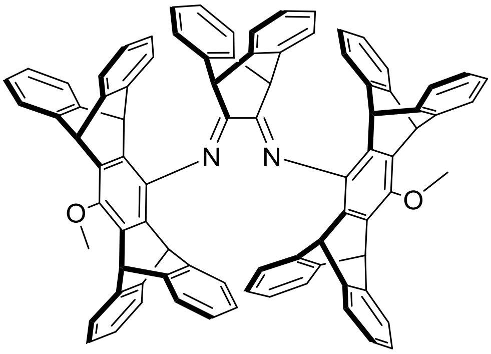

Preparation of Ligand: A solution of 9,10-dihydro-9,10-ethanoanthracene-11,12-dione (500 mg, 2.12 mmol), pentiptycene aminoanisole (2.5 g, 5.29 mmol) and *p*-toluenesulfonic acid (5 mg) in toluene (100 mL) was refluxed with Dean-stark trap for 3 days, the solvent was partially evaporated under reduced pressure until the formation of a yellow solid, and the remaining solution was diluted in ethanol (300 mL). The yellow solid was isolated by filtration, washed three times by 20 mL ethanol and dried under high vacuum (1.46 g, 60.2% yield). This method was similar to our previous study.[15]

^{1}H NMR (500 MHz, 298 K, DMSO-d_6, 2.50 ppm): δ =7.65-7.55 (m, 4H, aryl-*H*), 7.40-7.50 (m, 8H, aryl-*H*), 7.31-7.26 (m, 4H, aryl-*H*), 7.25-7.19 (m, 4H, aryl-*H*), 7.16-7.10 (m, 8H, aryl-*H*), 7.08-7.00 (m, 4H, aryl-*H*), 6.99-6.89 (m, 8H, aryl- *H*), 5.94 (s, 4H, C*H*Ph_2), 5.11 (s, 4H, C*H*Ph_2), 5.09 (s, 2H, C*H*Ph_2), 4.01 (s, 6H, OCH_3) ppm.

Elemental analysis: Anal. Calcd for $C_{86}H_{56}N_2O_2$: C, 89.87; H, 4.91; N, 2.44. Found: C, 89.99; H, 4.97; N, 2.36.

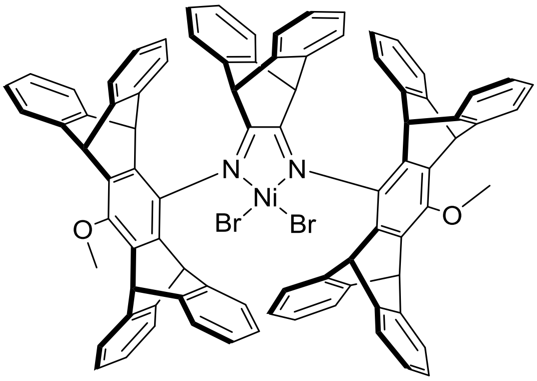

Preparation of ipty-Ni: To a solution of ligand which was reported by us previously [15] (200 mg, 0.174 mmol) in dry dichloromethane (25 mL) was added 53.8 mg (0.174 mmol) of $NiBr_2$(DME) (DME = 1,2-dimethoxyethane). After stirring the mixture for 3 days at room temperature, the solvent was evaporated under reduced pressure, the desired compound could be isolated from repeatedly recrystallized from n-hexane and dichloromethane. The pure compound was obtained as an orange solid. (190 mg, 79.8% yield)
MALDI-TOF-MS (m/z): 1285.29 $[M-Br]^+$
Elemental analysis: Anal. Calcd for $C_{86}H_{56}Br_2N_2NiO_2$: C, 75.51; H, 4.13; N, 2.05. Found: C, 75.80; H, 4.18; N, 2.00.

General Procedure for the Polymerization

A general procedure for ethylene polymerization using Ni catalyst
In a typical experiment, a 350 mL glass pressure reactor connected with a high pressure gas line was firstly dried at 90 ℃ under vacuum for at least 1 h. The reactor was then adjusted to the desired polymerization temperature. 98 mL of toluene and co-catalyst was added to the reactor under N_2 atmosphere, then the desired amount of Ni catalyst in 2 mL of CH_2Cl_2 was injected into the polymerization system via syringe. With a rapid stirring, the reactor was pressurized and maintained at specified pressure of ethylene. After the specified time, the pressure reactor was vented and the polymerization was quenched via the addition of 100 mL acidic EtOH (5% HCl in EtOH) and dried in a vacuum oven to constant weight.

Supplementary Tables

Supplementary Table 1. Study on the time effect of ethylene polymerization with ipty-Ni[a]

entry	*T* (°C)	T (min)	yield (g)	act. (10^6)[b]
1	30	5	0.11	1.32
2	30	10	0.34	2.04
3	30	20	0.90	2.70
4	30	30	1.24	2.48
5	30	45	2.05	2.73
6	90	5	0.16	1.92
7	90	10	0.43	2.58
8	90	20	0.51	1.53
9	90	30	0.60	1.20

[a] Reaction conditions: ipty-Ni catalyst (1 μmol), MMAO (500 eq.), toluene/CH_2Cl_2 (98 mL/2 mL), ethylene pressure (8 bar), all entries are based on at least two runs, unless noted otherwise. [b] Activity is in unit of g mol^{-1} h^{-1}.

Supplementary Table 2. Crystallographic data for ipty-Ni.

	ipty-Ni
Formula	$C_{86}H_{56}Br_2N_2NiO_2$
Formula weight	1367.85
Crystal dimensions (mm^3)	0.30 × 0.28 × 0.20
Crystal system	monoclinic
Space group	P 1 21/m 1
a (Å)	12.5340(14)
b (Å)	19.232(2)
c (Å)	16.085(2)
α (°)	90
β (°)	111.704(8)
γ (°)	90
Volume (Å^3)	3602.5(8)
Z	2
T (K)	173(2)
D_{calcd} (g cm^{-3})	1.261
μ (mm^{-1})	2.059
F (000)	1400
No. of rflns. collected	17682
No. of indep. rflns. /R_{int}	5244 / 0.1321
No. of obsd. rflns. [$I_0 > 2\sigma(I_0)$]	3448
Data / restraints / parameters	5244 / 0 / 424
R_1 / wR_2 [$I_0 > 2\sigma(I_0)$]	0.0837 / 0.1818
R_1 / wR_2 (all data)	0.1173 / 0.1992
GOF (on F^2)	1.138
Largest diff. peak and hole (e Å^{-3})	1.186 / -0.619
CCDC No.	1974098

Supplementary Figures

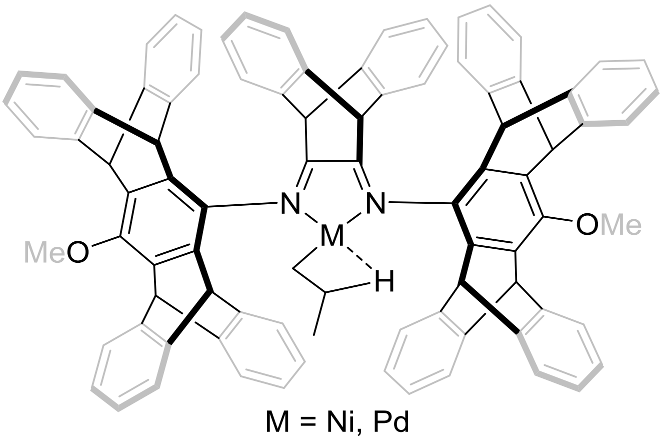

M = Ni, Pd

Supplementary Figure 1. The structure of complex $1_{\beta\text{-T}}$.

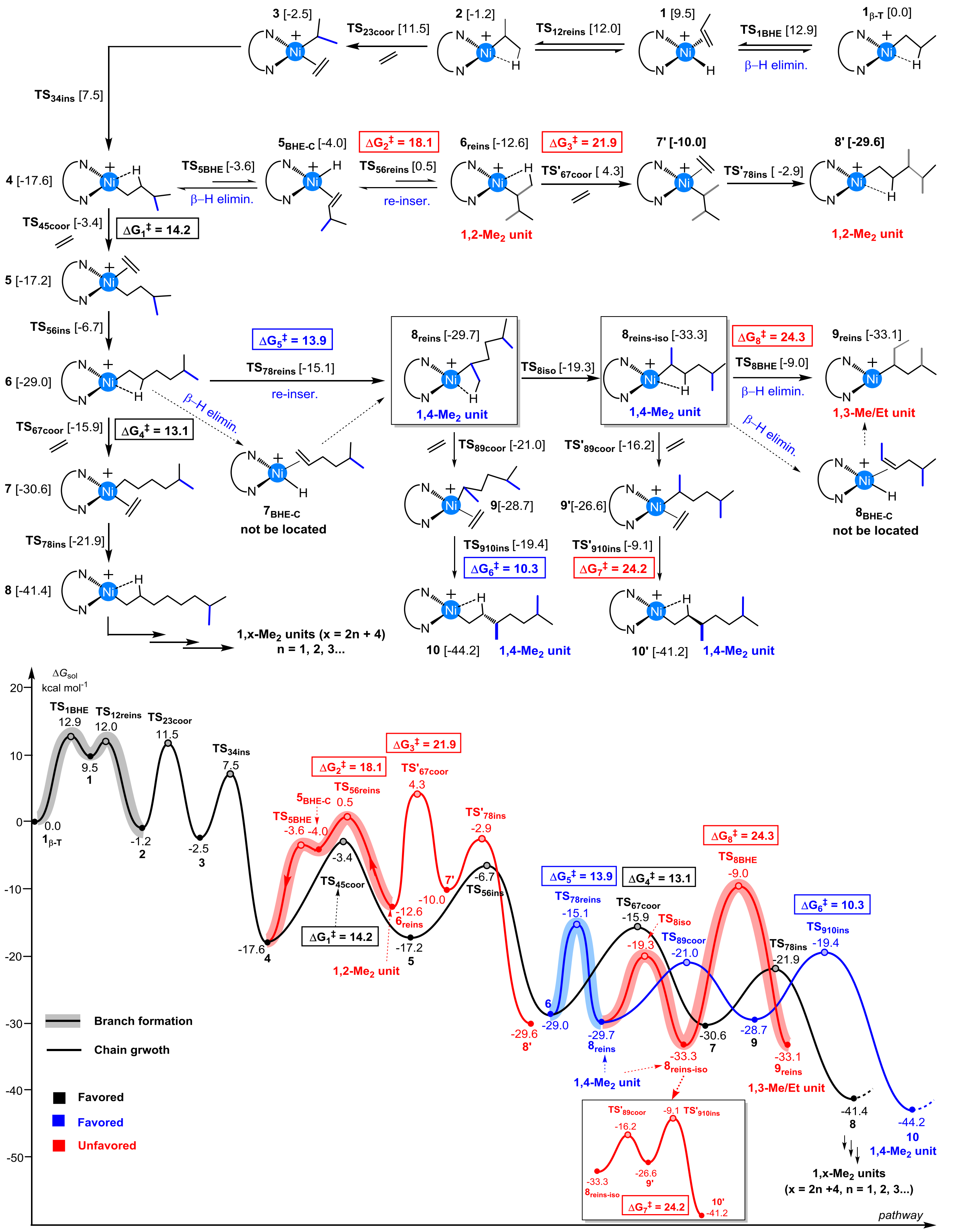

Supplementary Figure 2. Mechanism for the Formation of Branch Generated by **ipty-Ni** from the DFT simulation.

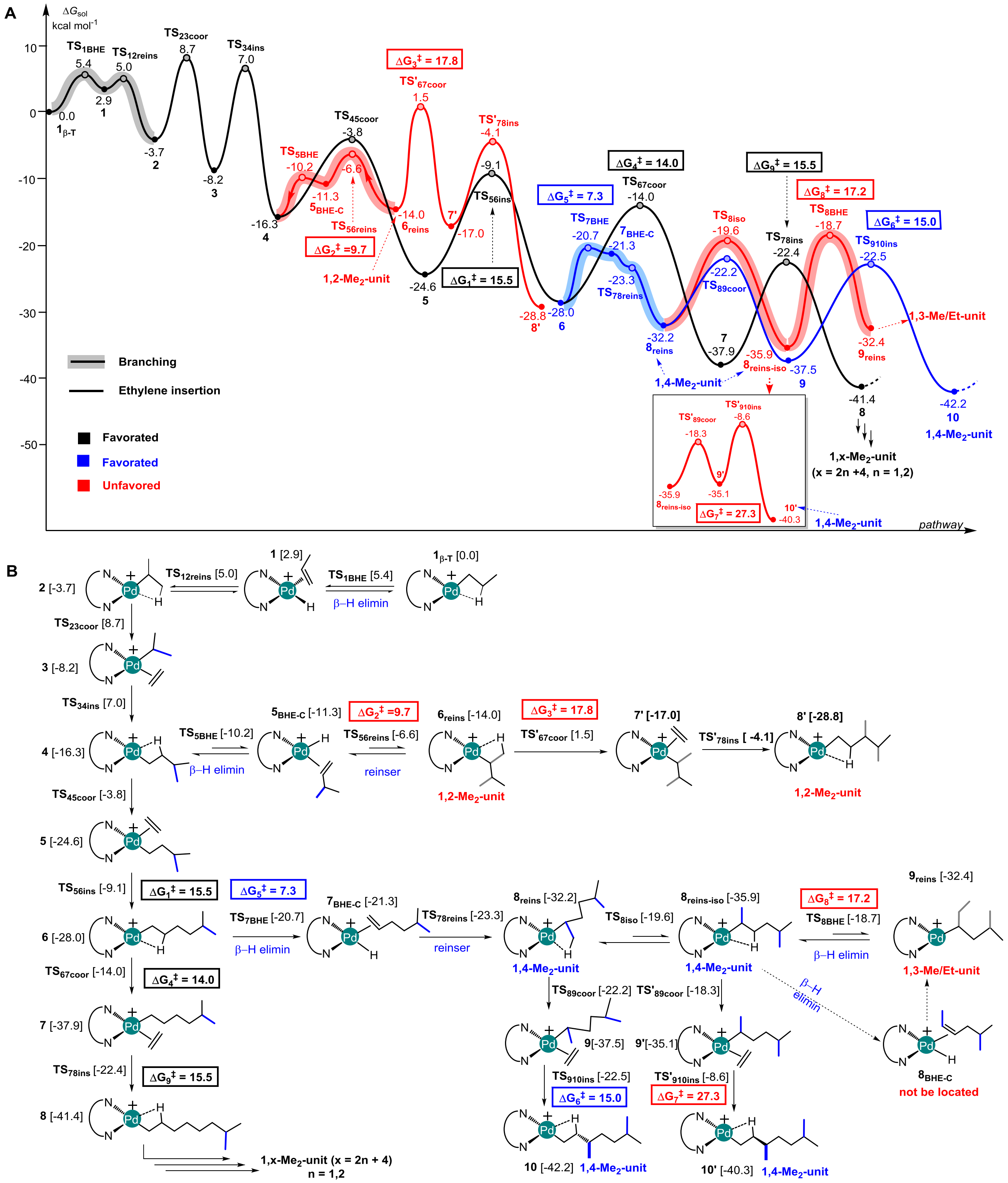

Supplementary Figure 3. Mechanism for the Formation of Branch Generated by **ipty-Pd** from the DFT simulation.

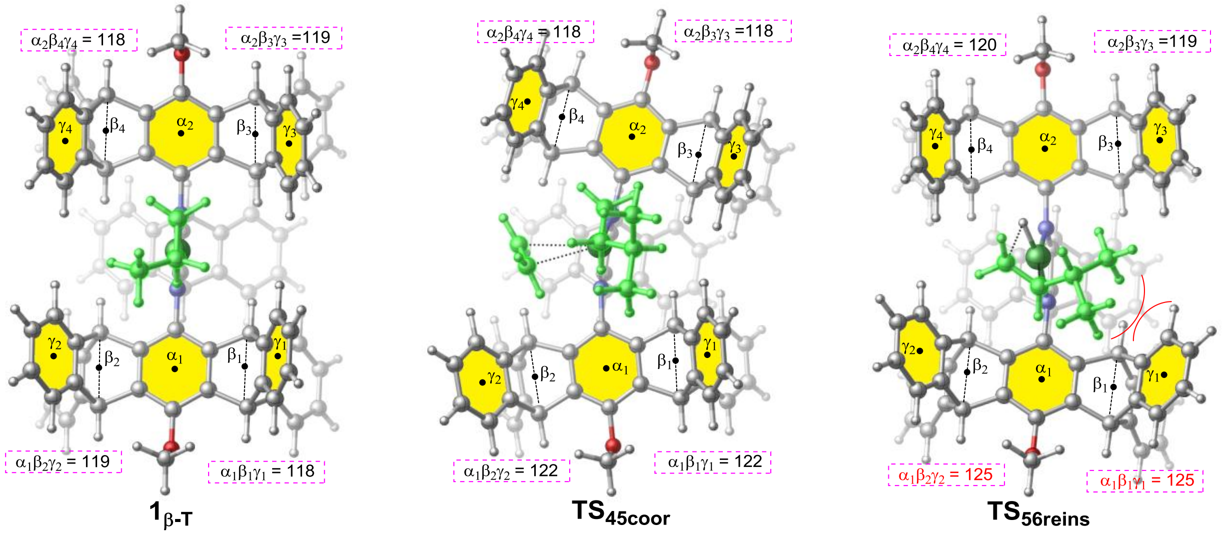

Supplementary Figure 4. Optimized geometrics (dihedral angles in degree) of $\mathbf{1}_{\beta\text{-T}}$, $\mathbf{TS}_{\mathbf{45coor}}$, and $\mathbf{TS}_{\mathbf{56reins}}$.

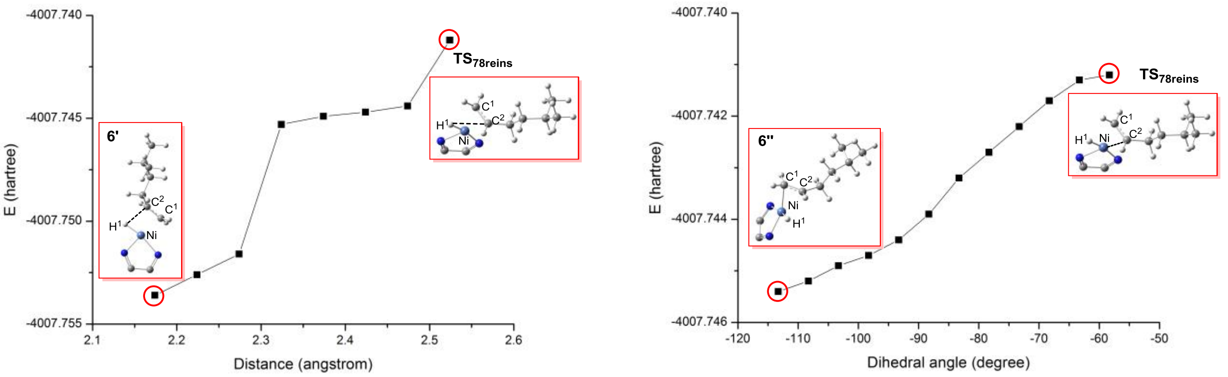

Supplementary Figure 5. Relaxed scan of potential energy surface (PES) for the distance (angstrom) of C2 and H1 and dihedral angle (degree) ∠H1-Ni-C2-C1 in $\mathbf{TS}_{\mathbf{78reins}}$ at the DFT/BSI level. We can observe that the dihedral angle (degree) ∠H1-Ni-C2-C1 in $\mathbf{TS}_{\mathbf{78reins}}$ is about 60° and the $\mathbf{TS}_{\mathbf{78reins}}$ from its' geometry is very near to $\mathbf{8}_{\mathbf{reins}}$. So we just scan the distance of C2 and H1 and dihedral angle ∠H1-Ni-C2-C1 in $\mathbf{TS}_{\mathbf{78reins}}$ to the direction of **6**, and no other transition state and intermediate are found. In addition, we also confirm that all transition state structures are shown to connect the reactant and product on either side via intrinsic reaction coordinate (IRC) following. For example, the structure $\mathbf{TS}_{\mathbf{78reins}}$ definitely connecting the reactant **6** and product $\mathbf{8}_{\mathbf{reins}}$ is verified as shown in next Supplementary Figure 6.

IRC analysis for **TS$_{78reins}$**

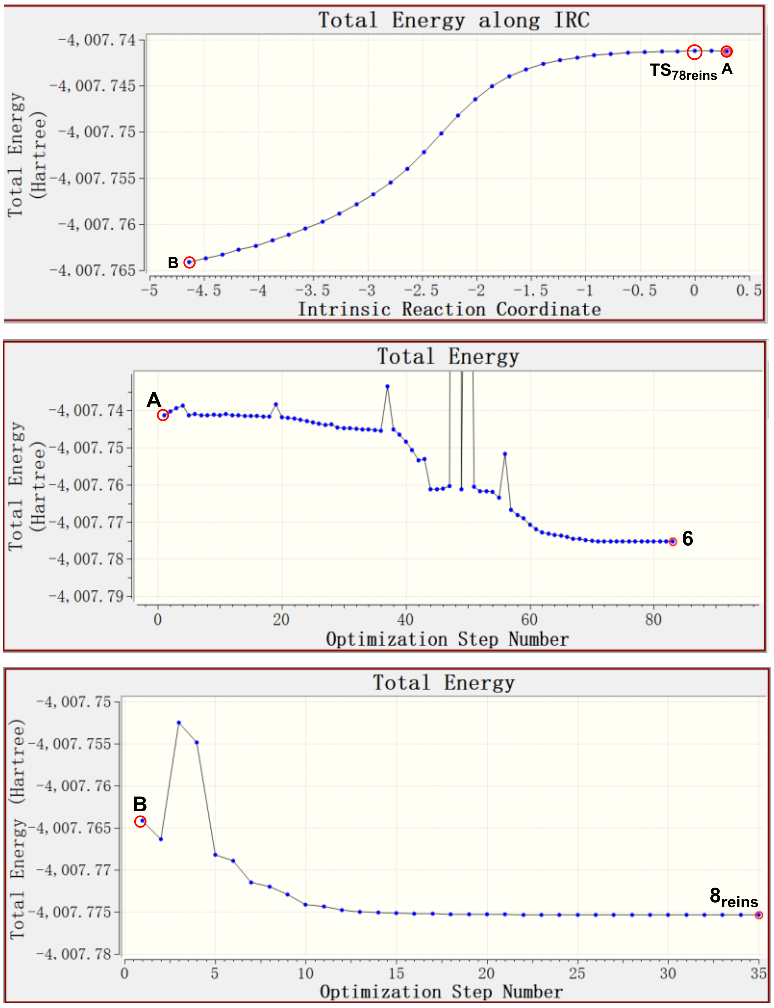

Supplementary Figure 6. IRC analysis for **TS$_{78reins}$** and optimized processes of the **6** and **8$_{reins}$**.

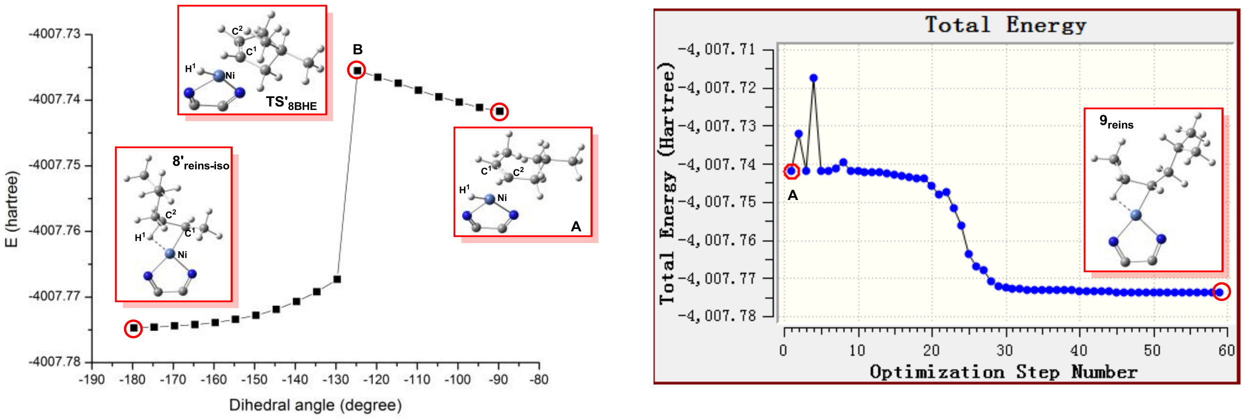

Supplementary Figure 7. Relaxed scan of potential energy surface (PES) for the dihedral angle (degree) ∠H1-Ni-C2-C1 and the optimization process of **9$_{reins}$** starting from **A** at the DFT/BSI level. For exploring the reaction pathway from of **8$_{reins-iso}$** and **9$_{reins}$**, we scan the dihedral angle (degree) ∠H1-Ni-C2-C1 and just one peak point is found. The optimized results confirm that the peak point **B** and the **A** point are optimized to give **TS$_{8BHE}$** and **9$_{reins}$**, respectively.

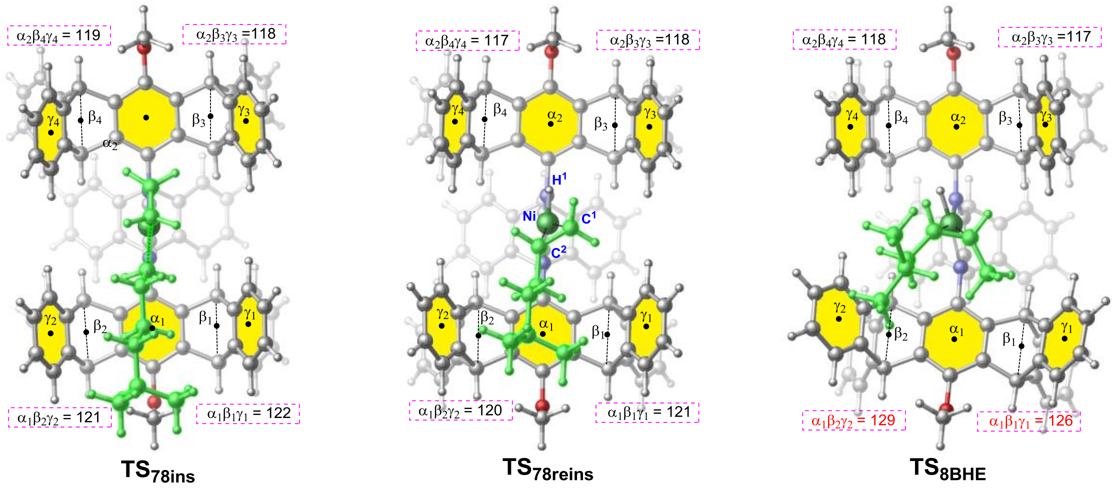

Supplementary Figure 8. Optimized geometrics (dihedral angles in degree) of **TS_{78ins}, $TS_{78reins}$,** and **TS_{8BHE}**. The optimized geometrics for TS_{78ins}, $TS_{78reins,}$ and TS_{8BHE} was comparatively analyzed. In TS_{8BHE}, the bigger steric repulsion between MH and phenyl groups, as suggested by the bigger dihetral angles (126/129° vs. 122/121° in TS_{78ins} and 121/120° in $TS_{78reins}$), could account for the less stability of TS_{8BHE} in comparison with TS_{78ins} and $TS_{78reins}$.

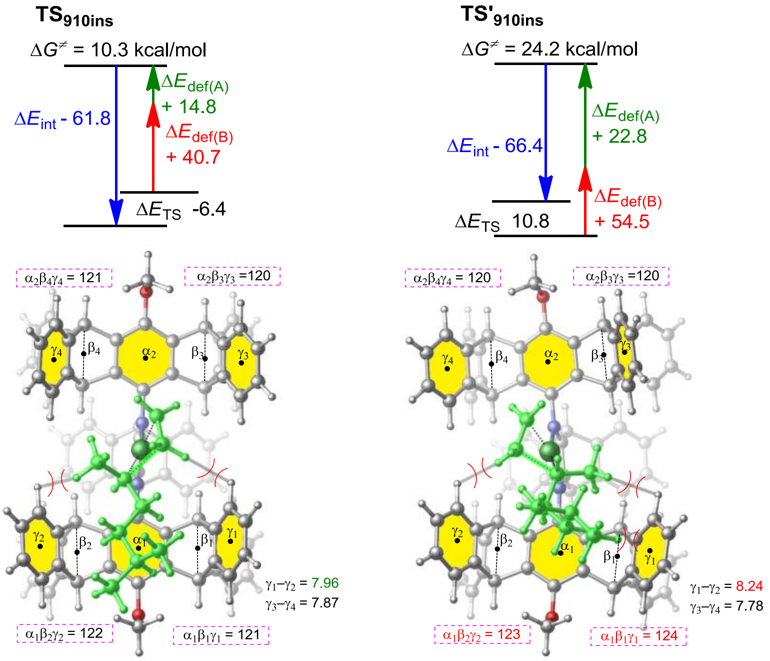

Supplementary Figure 9. Energy decomposition (ΔE in kcal mol^{-1}) and geometrical analyses (dihedral angles in degree and distance in Å) for **TS_{910ins}** and **TS'_{910ins}**. Further energy decomposition and structural analyses indicate that the less stability of TS'_{910ins} could be ascribed to the bigger repulsion of poly(ethylene) chain/ethylene monomer and auxiliary ligand, as suggested by a bigger deformation energy ($\Delta E_{def} = \Delta E_{def(A)} + \Delta E_{def(B)} = 77.3$ vs. 55.5 kcal mol^{-1} in TS_{910ins}), the bigger dihedral angles ($\angle\alpha_1\beta_1\gamma_1/\angle\alpha_1\beta_2\gamma_2$: 124°/123° vs. 121/122 in TS_{910ins}) and a farther distance (γ_1–γ_2 = 8.24 vs. 7.96 Å in TS_{910ins}) between two phenyl centers of ligand.

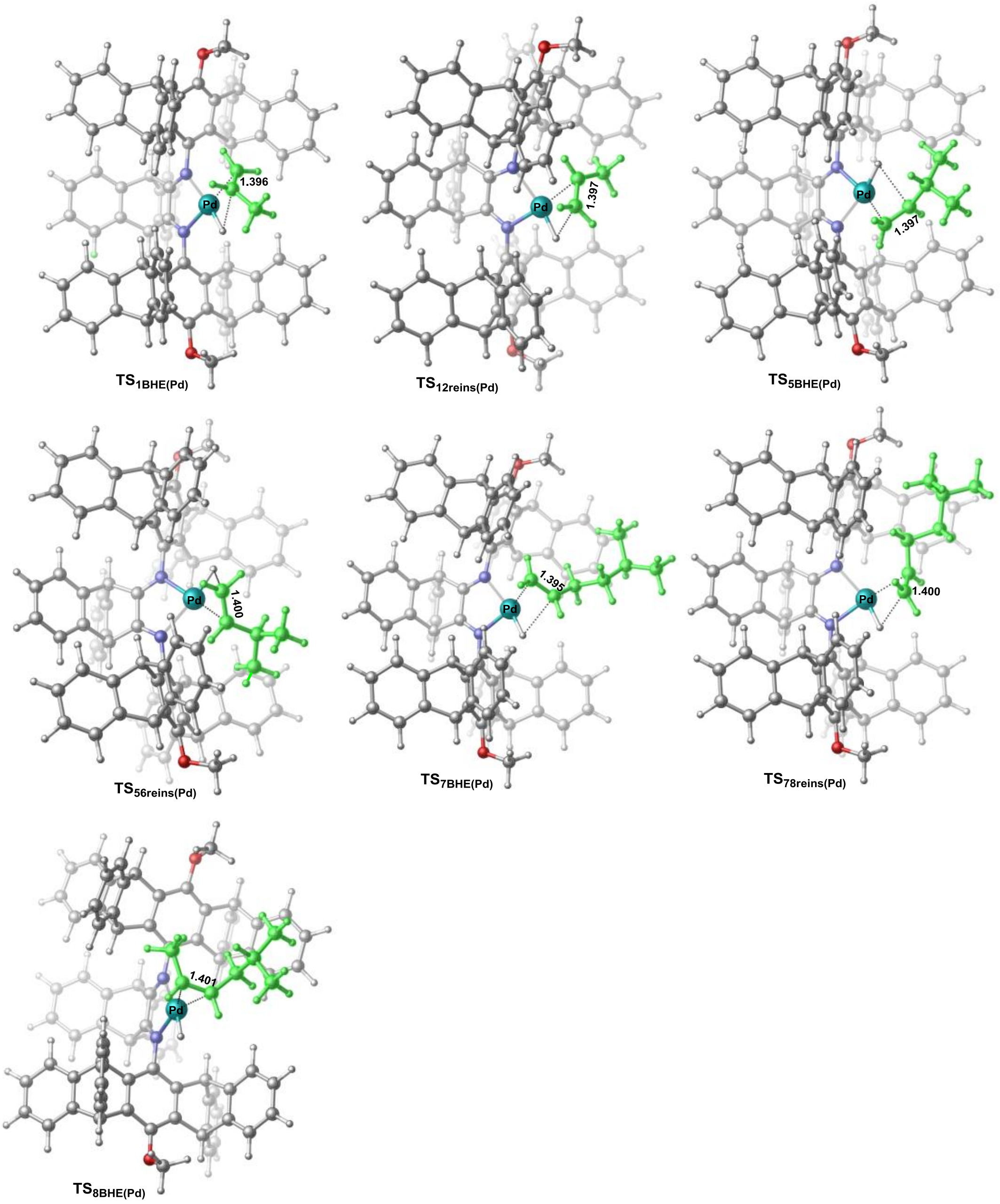

Supplementary Figure 10. Optimized geometrics (distance in Å) involved in branching process of **ipty-Pd** system.

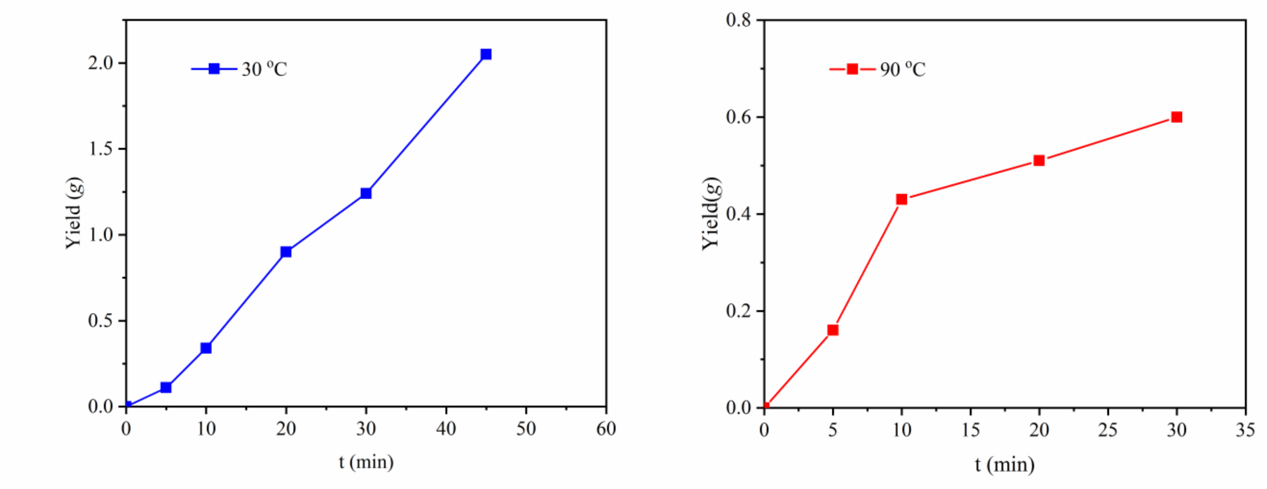

Supplementary Figure 11. Plots of yield of polyethylenes generated vs. reaction time with **ipty-Ni** at different temperatures in Supplementary Table 1.

^{1}H, ^{13}C, ^{1}H-^{1}H COSY NMR of ipty-Ni.

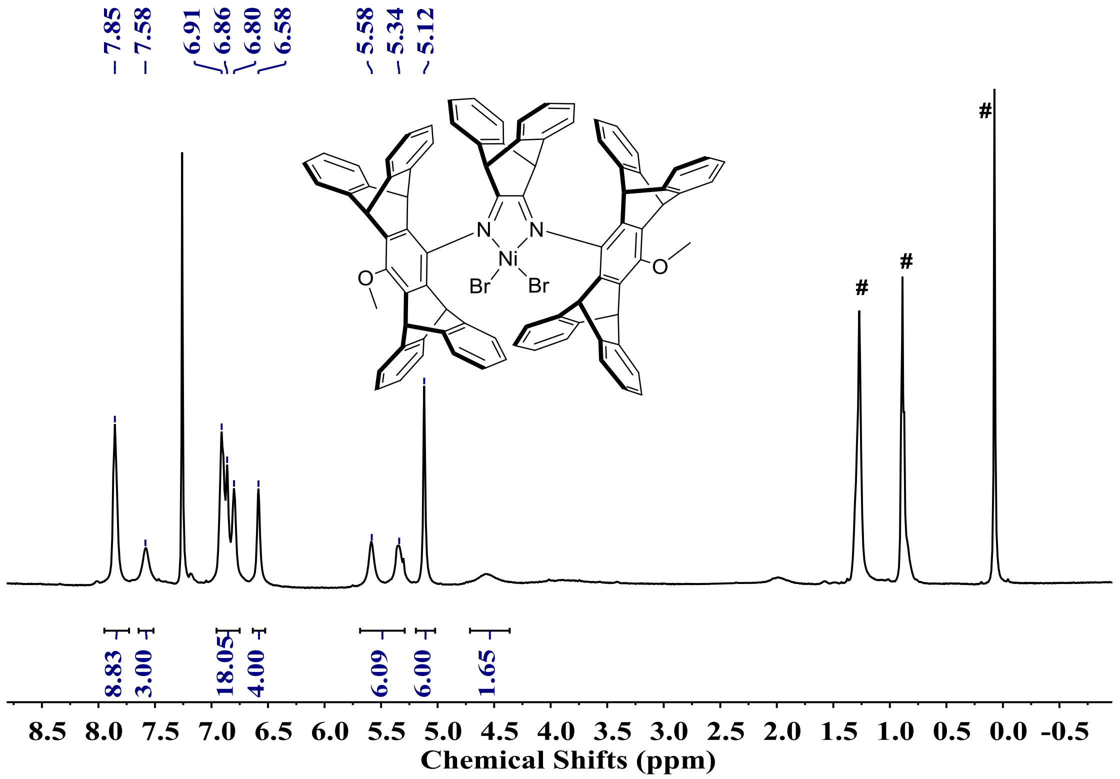

Supplementary Figure 12. ^{1}H NMR spectrum (298K, $CDCl_3$) of **ipty-Ni**.

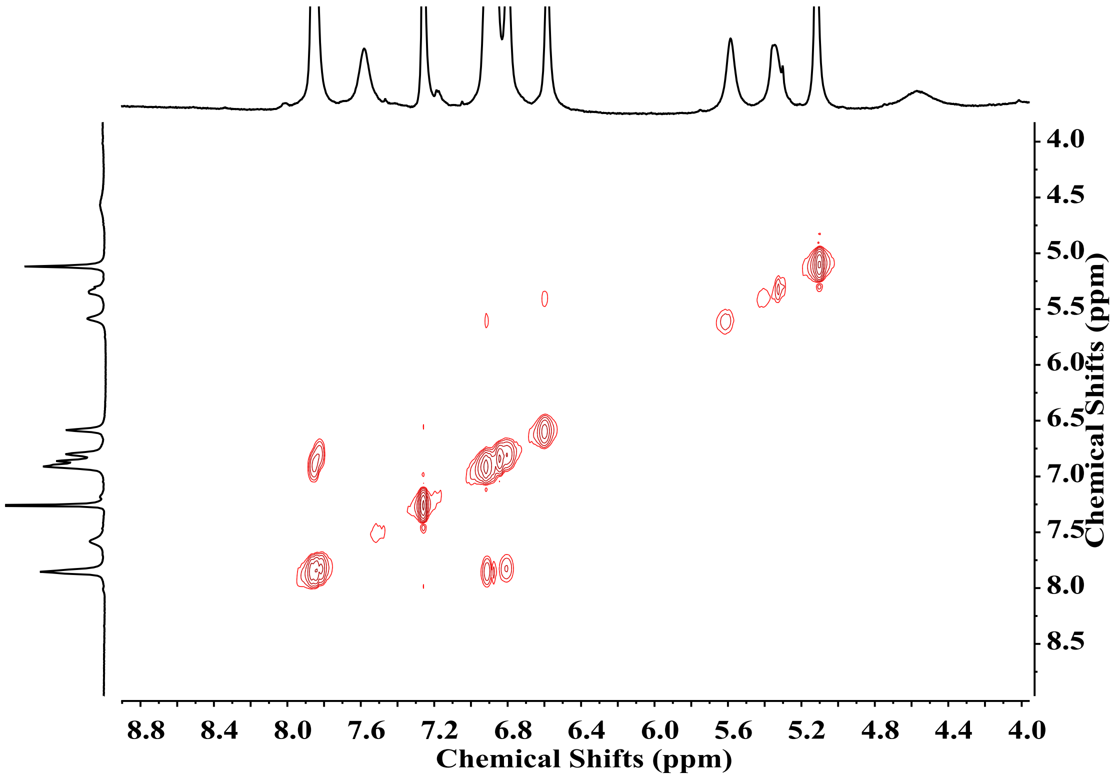

Supplementary Figure 13. ^1H-^1H COSY NMR spectrum (298K, $CDCl_3$) of **ipty-Ni**.

¹H NMR of Polymers.

Branches = Me groups / 1000 C = $\frac{2\times I_{Me}}{3\times I_{tot}}\times 1000 = \frac{2\times 3}{3\times(16.86+3)}\times 1000 = 101$

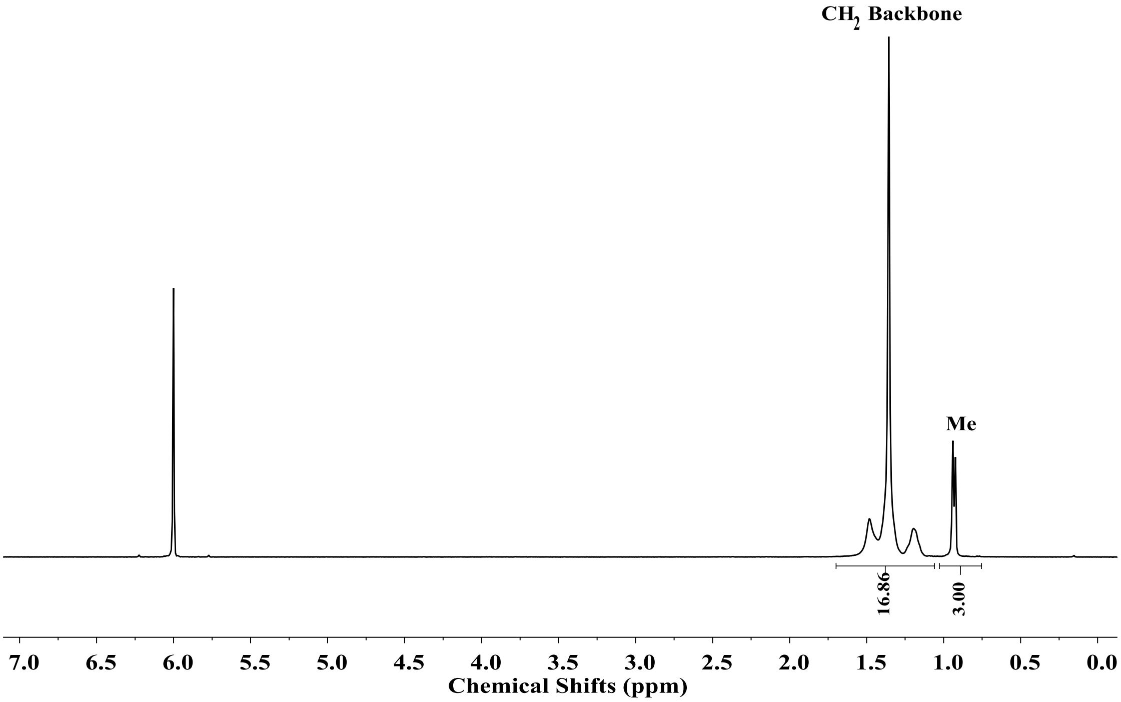

Supplementary Figure 14. ¹H NMR spectrum (400 MHz, $C_2D_2Cl_4$, 110 ℃) of the polyethylene generated by **ipty-Ni** from table 1, entry 1 and table 2, entry 1.

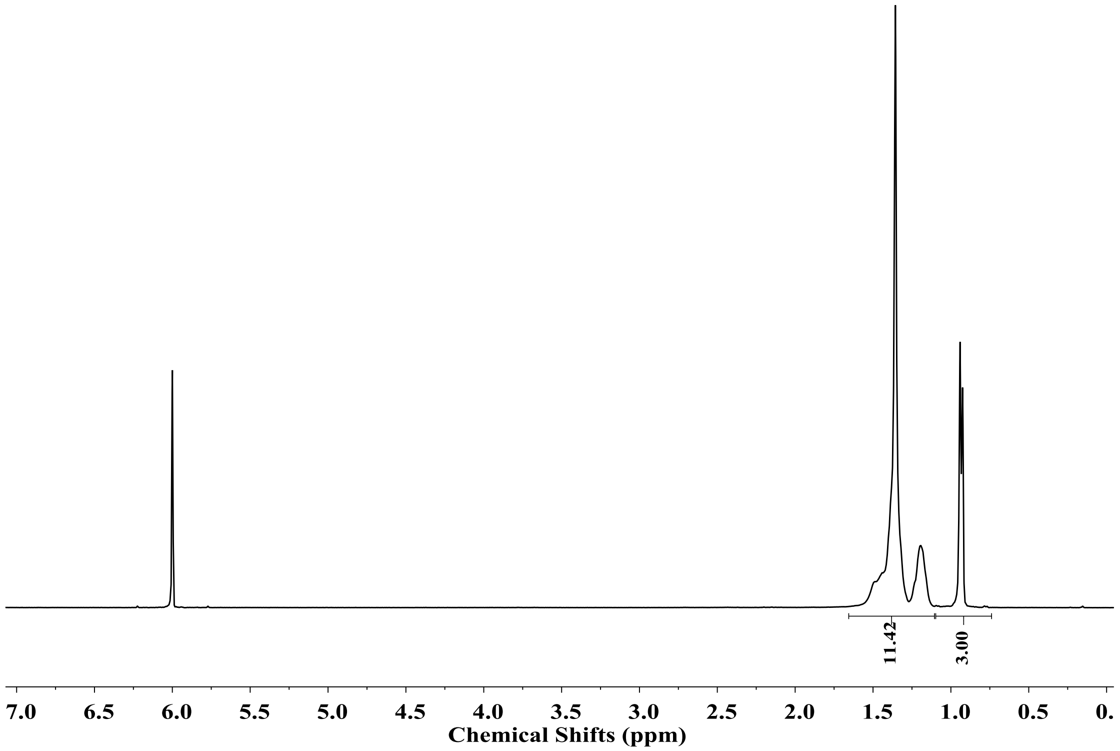

Supplementary Figure 15. ¹H NMR spectrum (400 MHz, $C_2D_2Cl_4$, 110 ℃) of the polyethylene generated by **ipty-Ni** from table 1, entry 2 and table 2, entry 2.

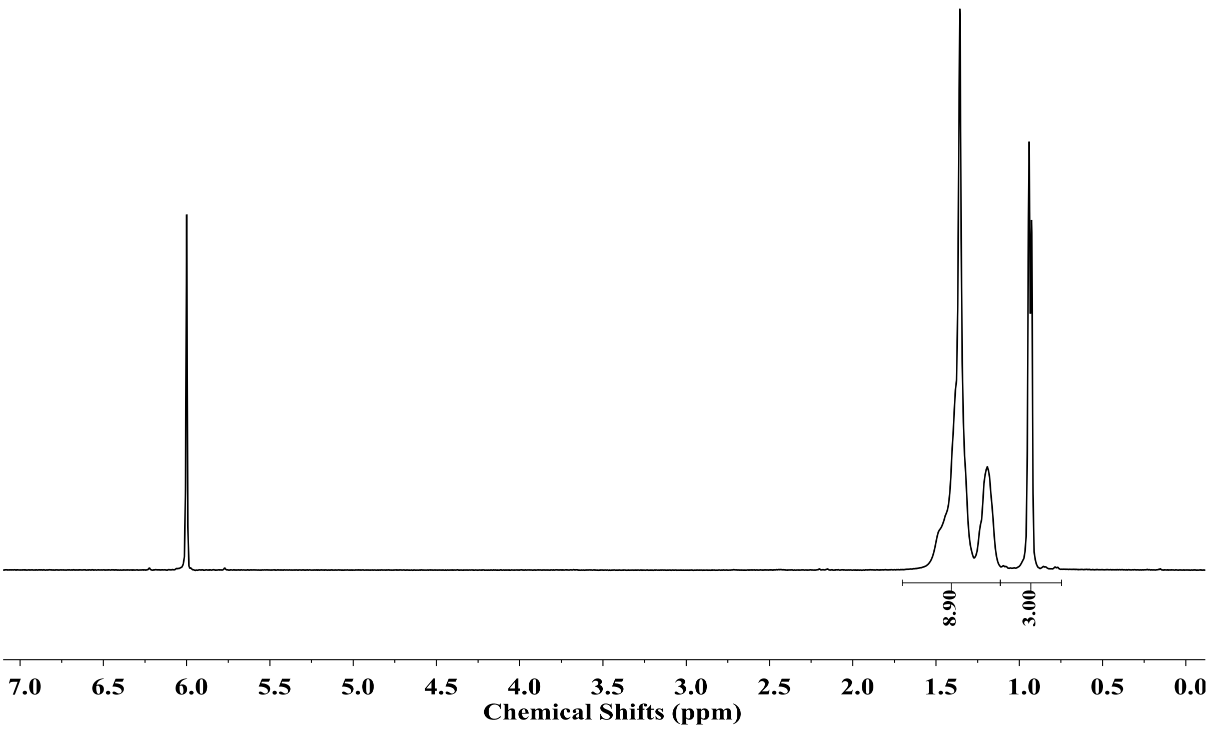

Supplementary Figure 16. ^{1}H NMR spectrum (400 MHz, $C_2D_2Cl_4$, 110 ℃) of the polyethylene generated by **ipty-Ni** from table 1, entry 3 and table 2, entry 3.

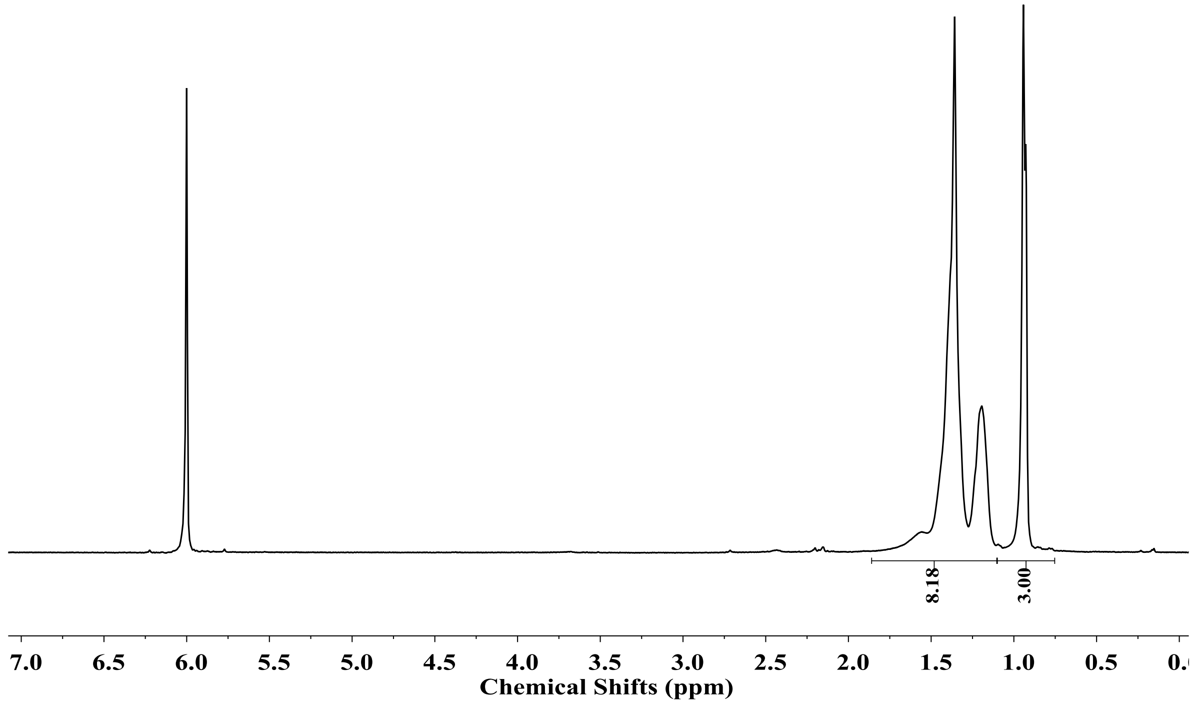

Supplementary Figure 17. ^{1}H NMR spectrum (400 MHz, $C_2D_2Cl_4$, 110 ℃) of the polyethylene generated by **ipty-Ni** from table 1, entry 4 and table 2, entry 4.

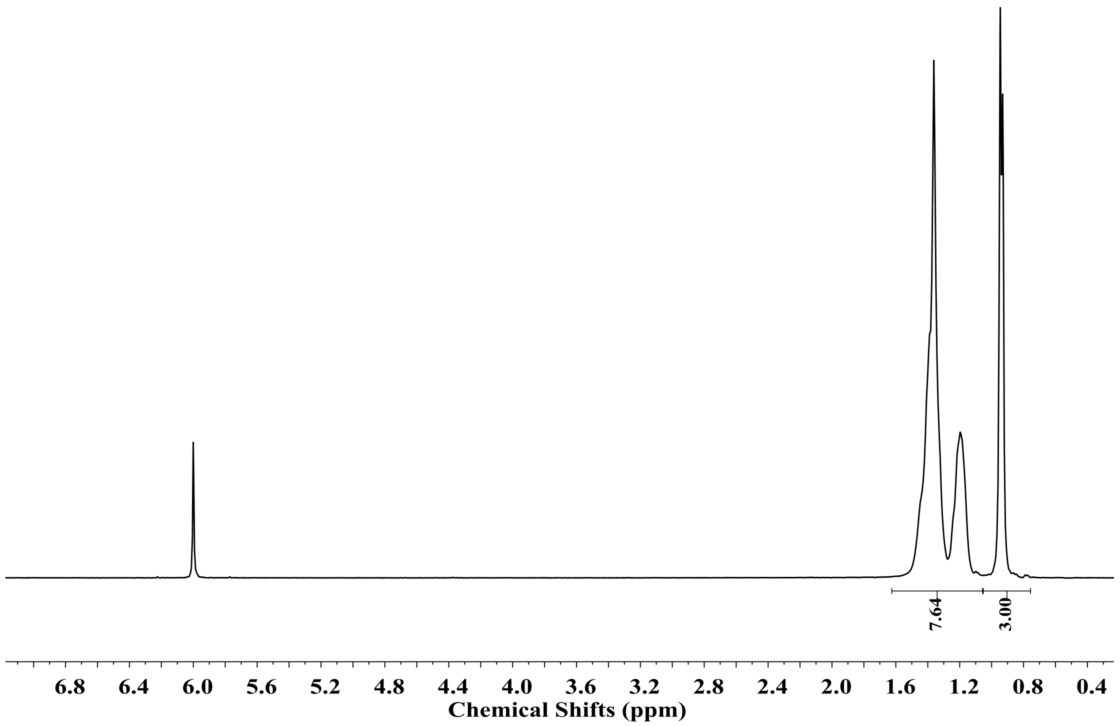

Supplementary Figure 18. ^{1}H NMR spectrum (400 MHz, $C_2D_2Cl_4$, 110 ℃) of the polyethylene generated by **ipty-Ni** from table 1, entry 5 and table 2, entry 5.

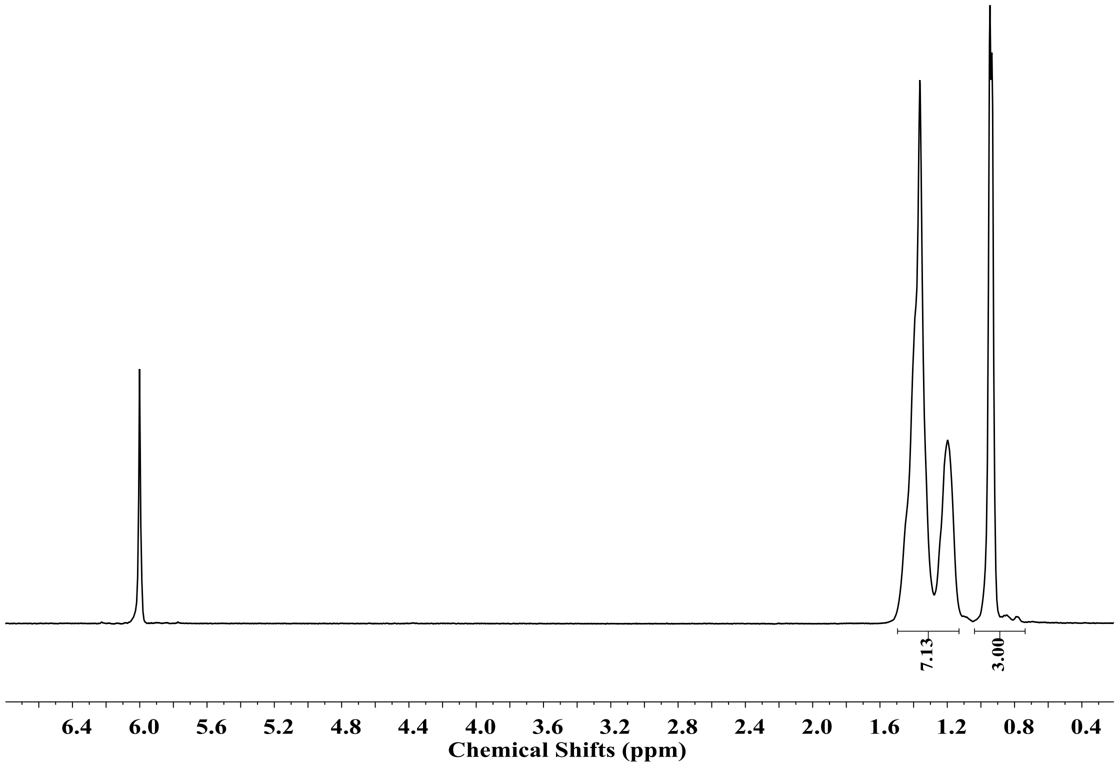

Supplementary Figure 19. ^{1}H NMR spectrum (400 MHz, $C_2D_2Cl_4$, 110 ℃) of the polyethylene generated by **ipty-Ni** from table 1, entry 6 and table 2, entry 6.

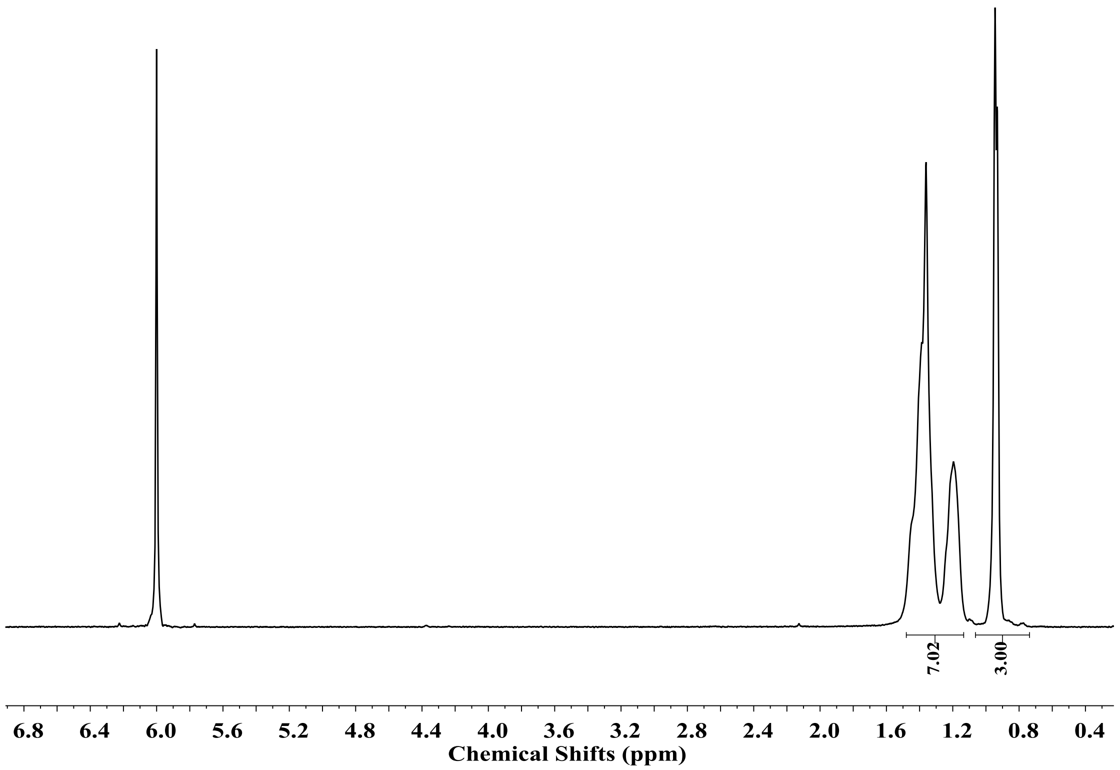

Supplementary Figure 20. ^{1}H NMR spectrum (400 MHz, $C_2D_2Cl_4$, 110 ℃) of the polyethylene generated by **ipty-Ni** from table 1, entry 7 and table 2, entry 7.

^{13}C NMR of Polymers and Branch Pattern Analysis.

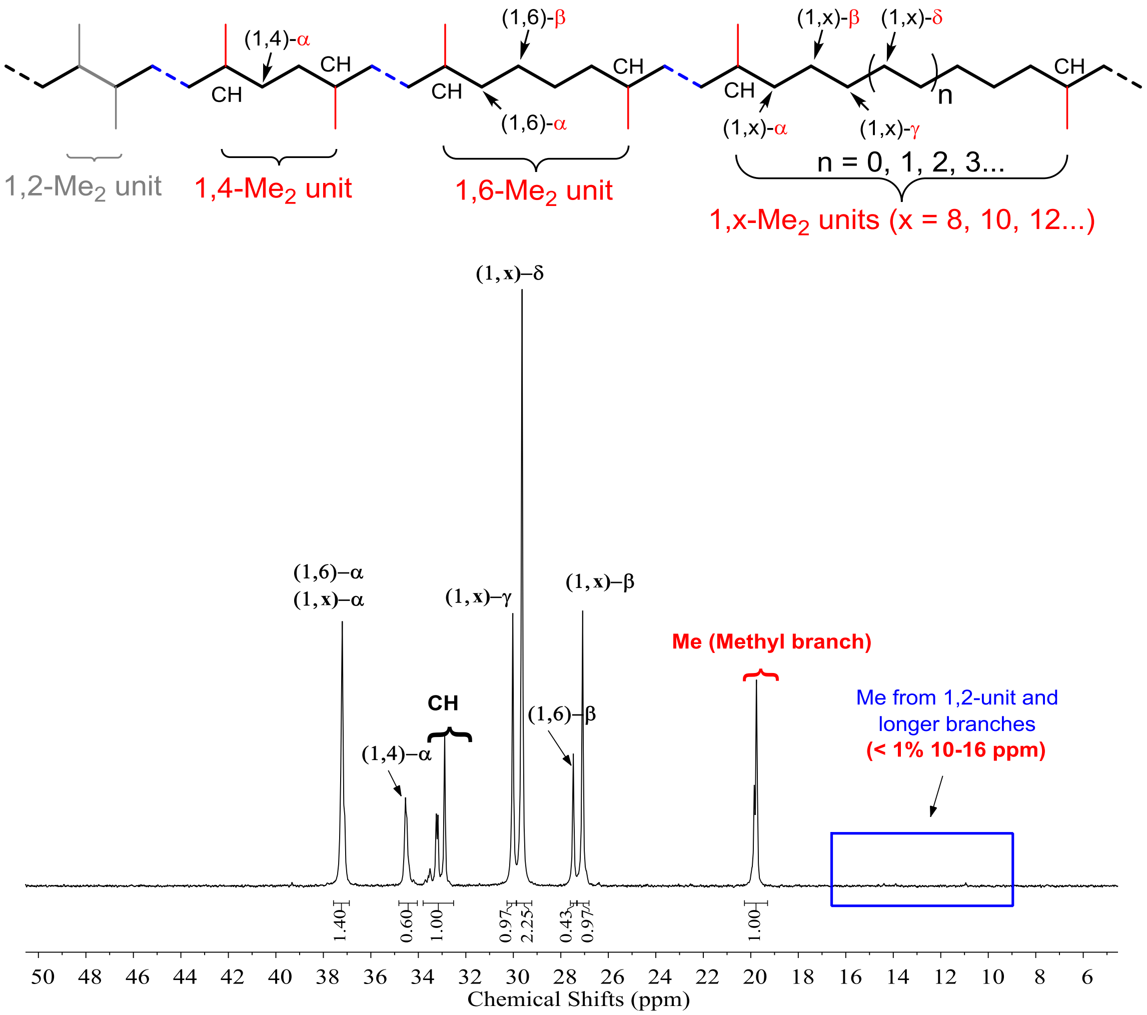

Supplementary Figure 21. ^{13}C NMR spectrum (400 MHz, $C_2D_2Cl_4$, 110 ℃) of the polyethylene generated by **ipty-Ni** from table 1, entry 1 and table 2, entry 1.

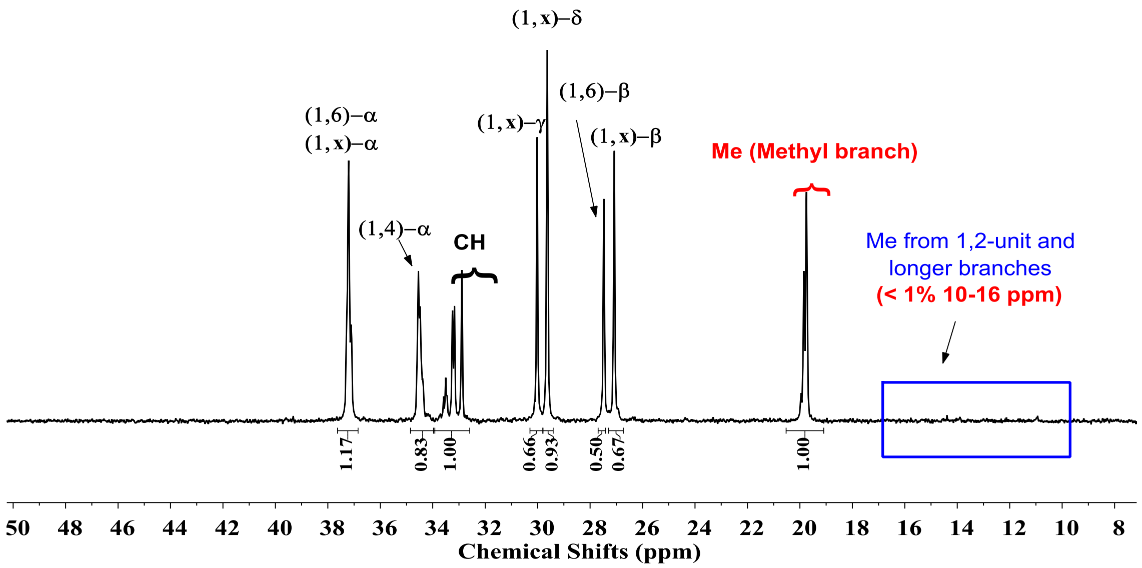

Supplementary Figure 22. ^{13}C NMR spectrum (400 MHz, $C_2D_2Cl_4$, 110 ℃) of the polyethylene generated by **ipty-Ni** from table 1, entry 2 and table 2, entry 2.

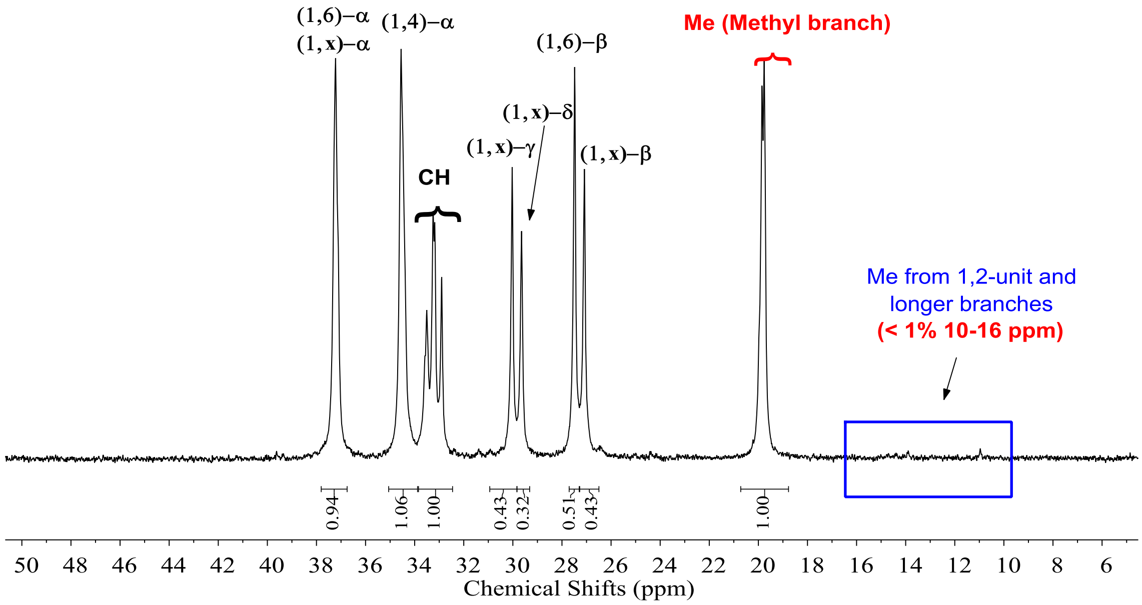

Supplementary Figure 23. ^{13}C NMR spectrum (400 MHz, $C_2D_2Cl_4$, 110 ℃) of the polyethylene generated by **ipty-Ni** from table 1, entry 3 and table 2, entry 3.

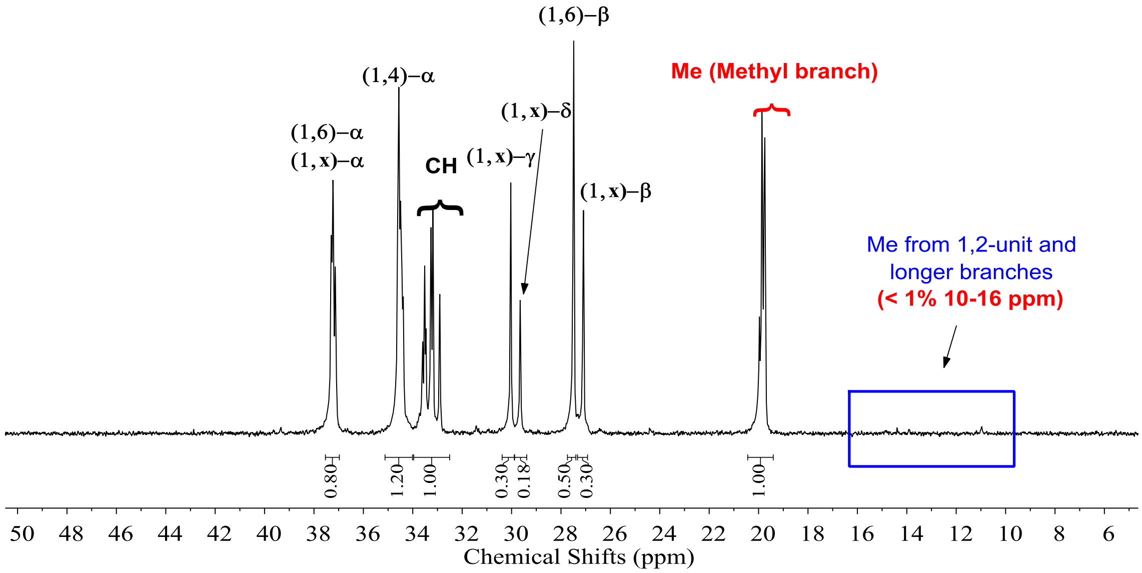

Supplementary Figure 24. ^{13}C NMR spectrum (400 MHz, $C_2D_2Cl_4$, 110 ℃) of the polyethylene generated by **ipty-Ni** from table 1, entry 4 and table 2, entry 4.

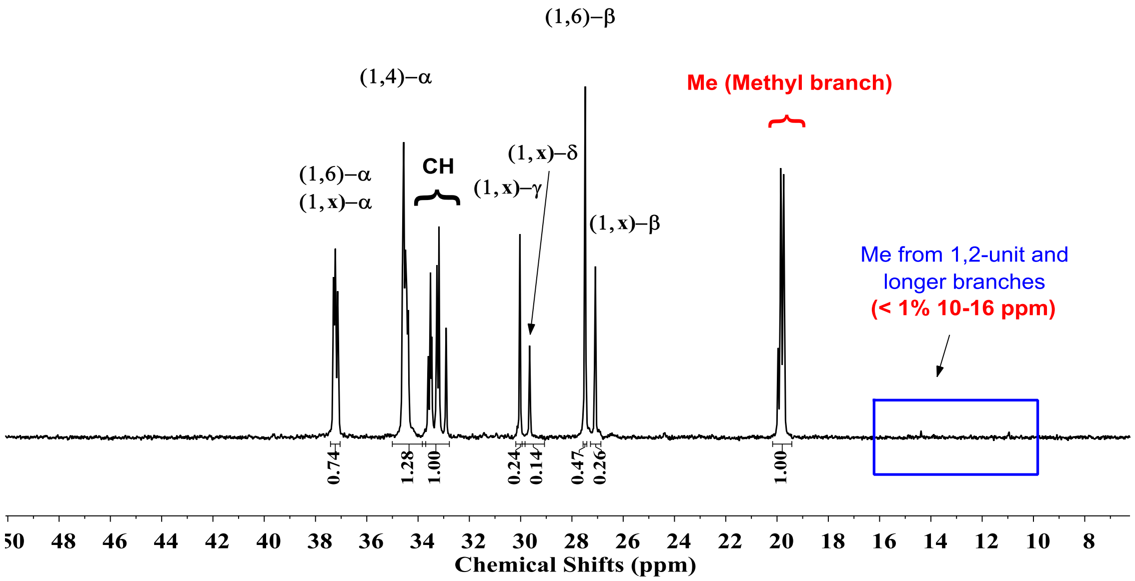

Supplementary Figure 25. ^{13}C NMR spectrum (400 MHz, $C_2D_2Cl_4$, 110 ℃) of the polyethylene generated by **ipty-Ni** from table 1, entry 5 and table 2, entry 5.

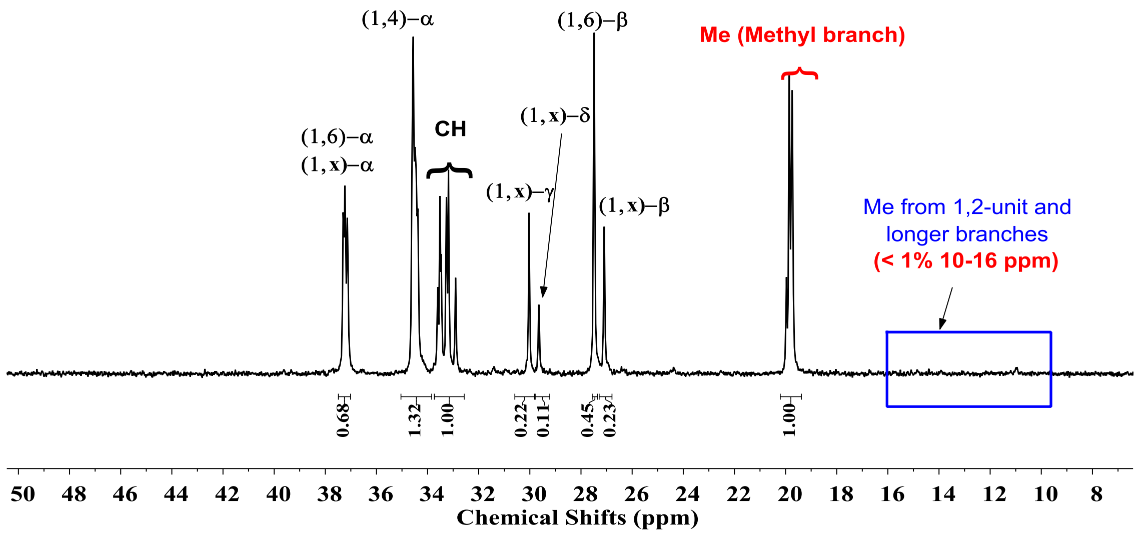

Supplementary Figure 26. ^{13}C NMR spectrum (400 MHz, $C_2D_2Cl_4$, 110 ℃) of the polyethylene generated by **ipty-Ni** from table 1, entry 6 and table 2, entry 6.

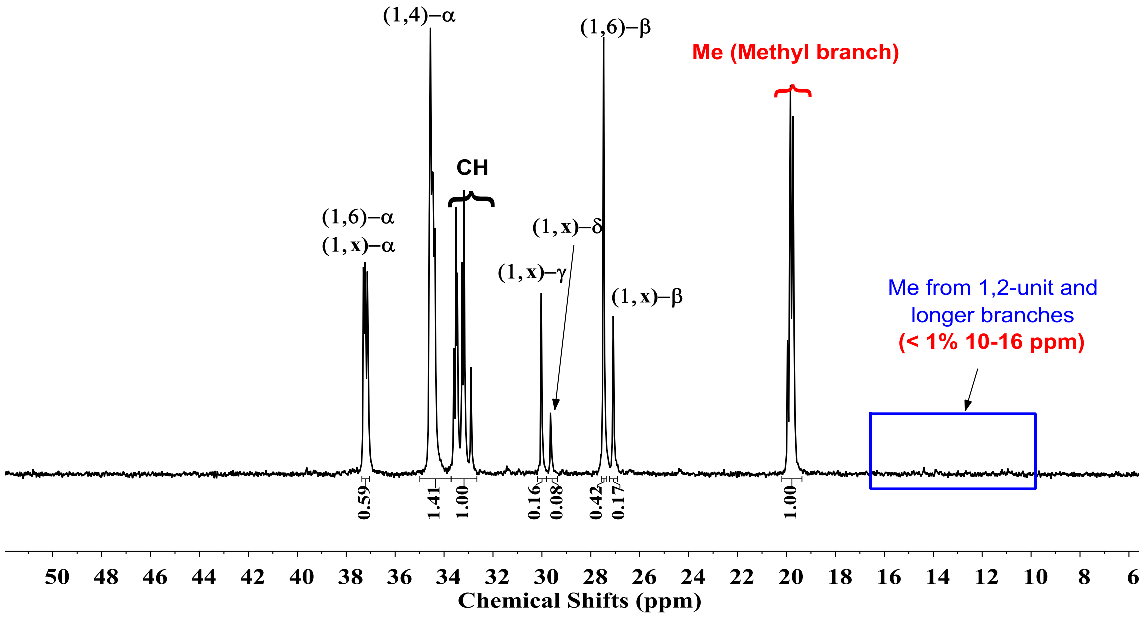

Supplementary Figure 27. ^{13}C NMR spectrum (400 MHz, $C_2D_2Cl_4$, 110 ℃) of the polyethylene generated by **ipty-Ni** from table 1, entry 7 and table 2, entry 7.

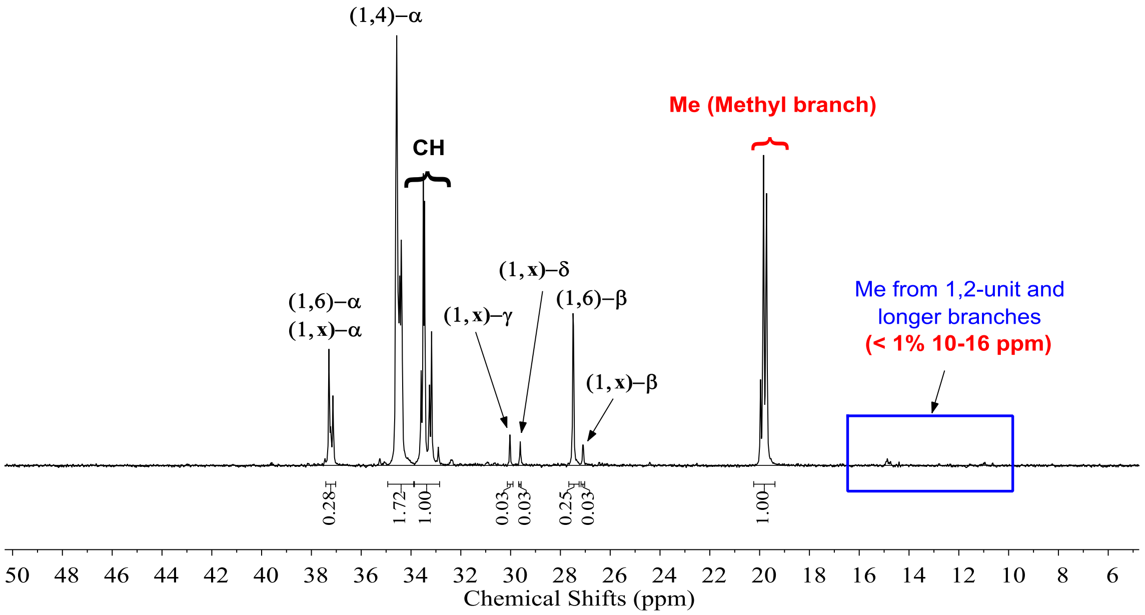

Supplementary Figure 28. ^{13}C NMR spectrum (400 MHz, $C_2D_2Cl_4$, 110 ℃) of the polyethylene generated by **ipty-Pd** from table 2, entry 8.

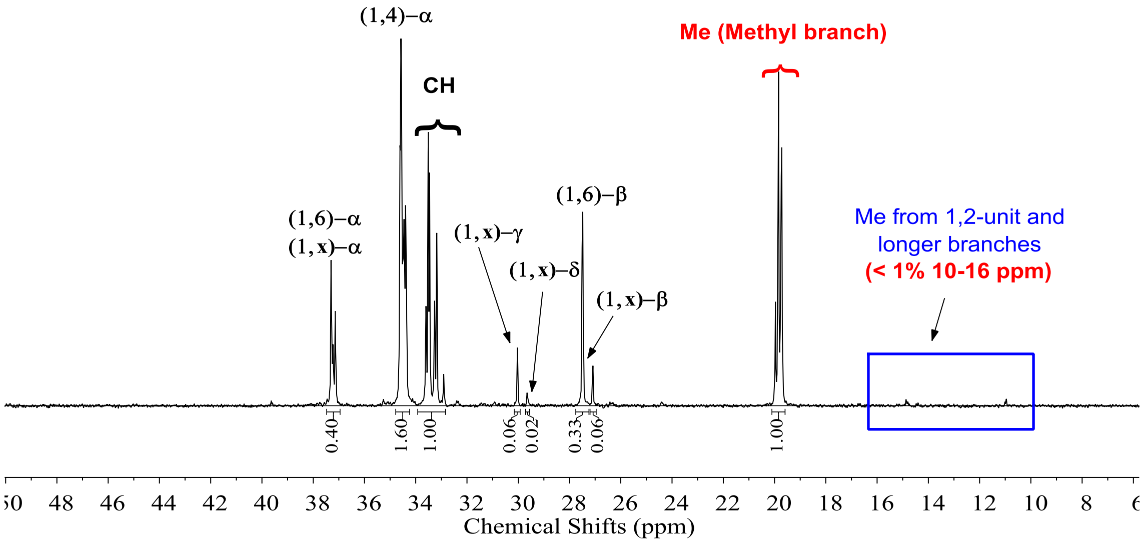

Supplementary Figure 29. ^{13}C NMR spectrum (400 MHz, $C_2D_2Cl_4$, 110 ℃) of the polyethylene generated by **ipty-Pd** from table 2, entry 9.

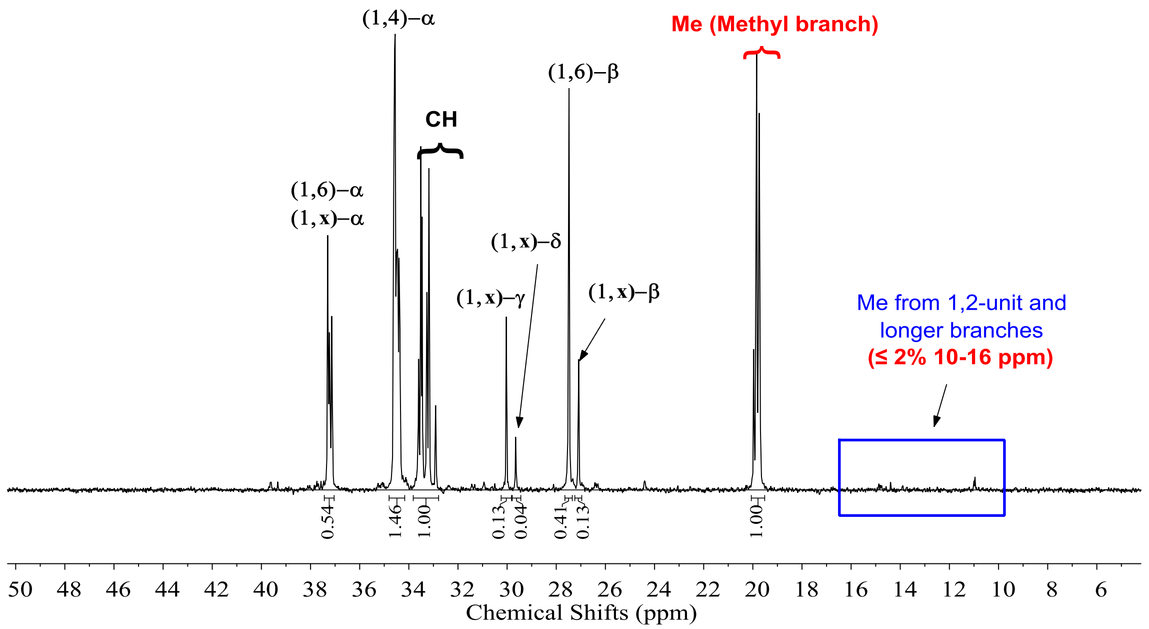

Supplementary Figure 30. ^{13}C NMR spectrum (400 MHz, $C_2D_2Cl_4$, 110 ℃) of the polyethylene generated by **ipty-Pd** from table 2, entry 10.

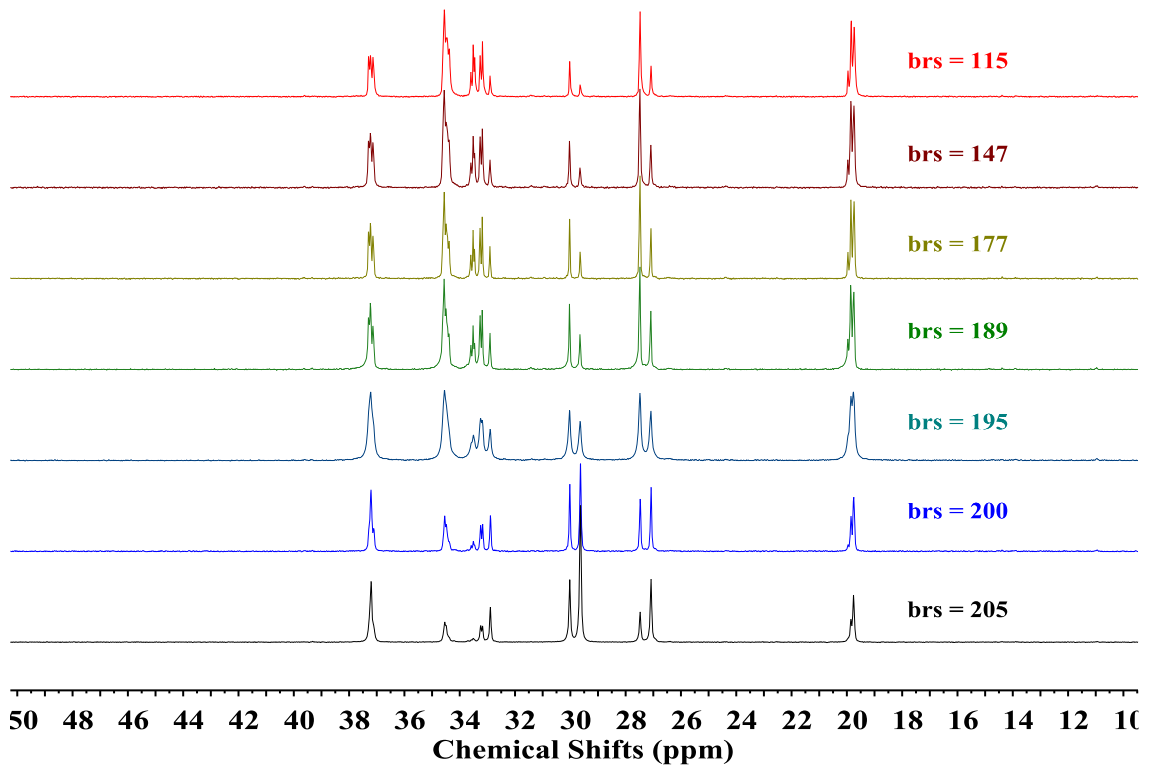

Supplementary Figure 31. ^{13}C NMR spectrum (100 MHz, $C_2D_2Cl_4$, 110 ℃) of the polyethylene generated by **ipty-Ni** from table 2, entry 1 to 7.

XRD Figures of Polymers

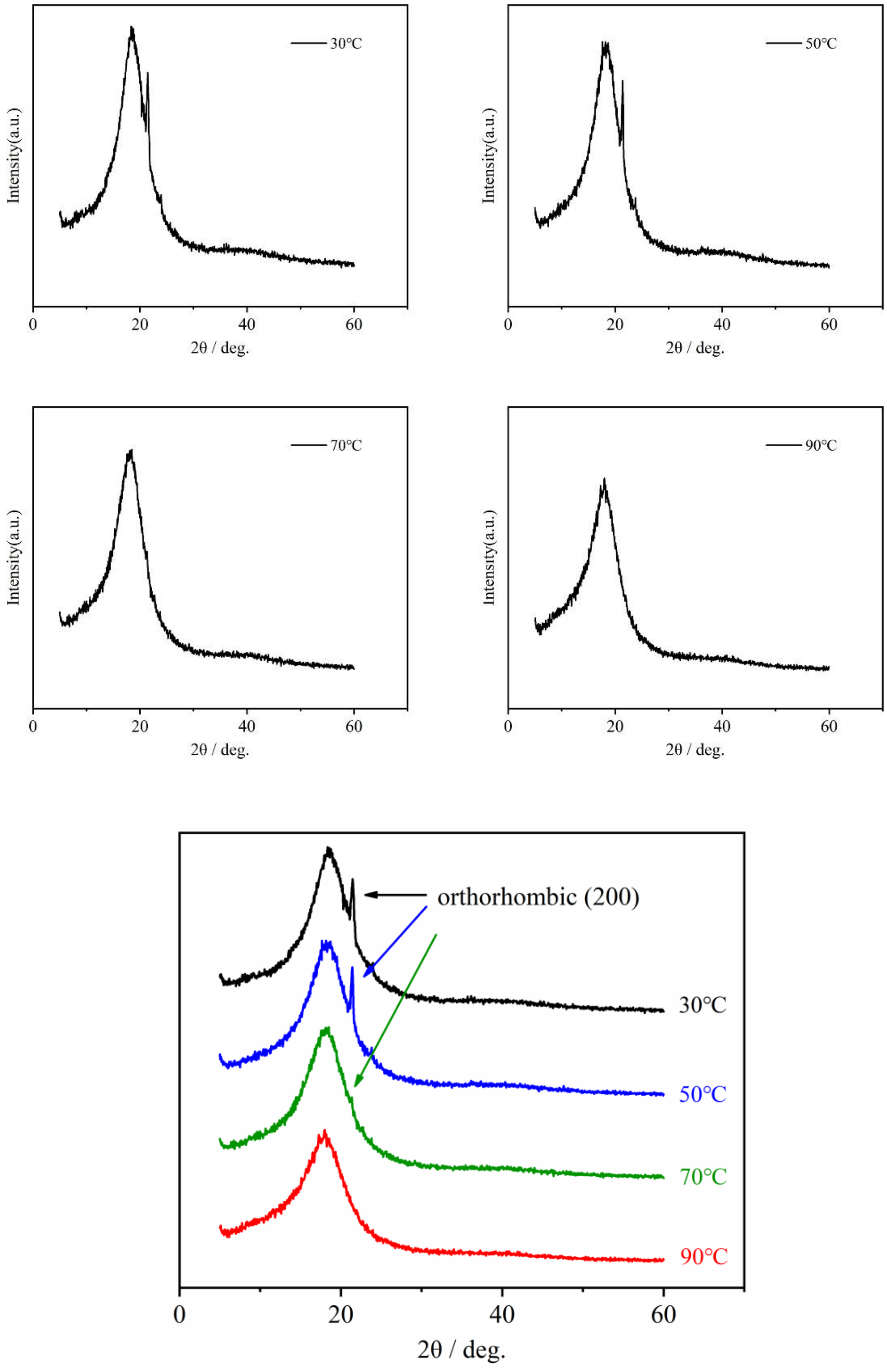

Supplementary Figure 32. XRD of the polymers generated by complex **ipty-Ni** from table 2, entry 1 to 4. Among them, partially crystalline polymers were obtained at temperature range of 30 °C to 70 °C.

GPC of Polymers

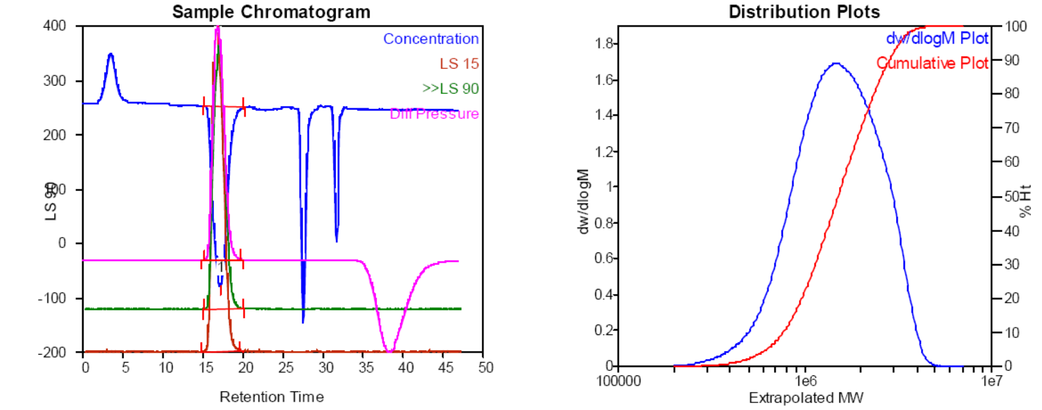

MW Averages

Peak No	Mp	Mn	Mw	Mz	Mz+1	Mv	PD
1	1474874	1287180	1662230	2048889	2410331	1572404	1.29137

Processed Peaks

Peak No	Name	Start RT (mins)	Max RT (mins)	End RT (mins)	Pk Height (mV)	% Height	Area (mV.secs)	% Area
1	Concentration	15.20	17.12	19.98	-21.2092	0	2511.37	100
2	LS 15	14.85	16.60	19.60	218.074	0	23433.7	100
3	LS 90	15.17	16.93	20.02	488.342	0	54235	100
4	Diff Pressure	15.10	16.80	19.65	820.084	0	87274.4	100

Supplementary Figure 33. GPC trace of the polymer from table 1, entry 1.

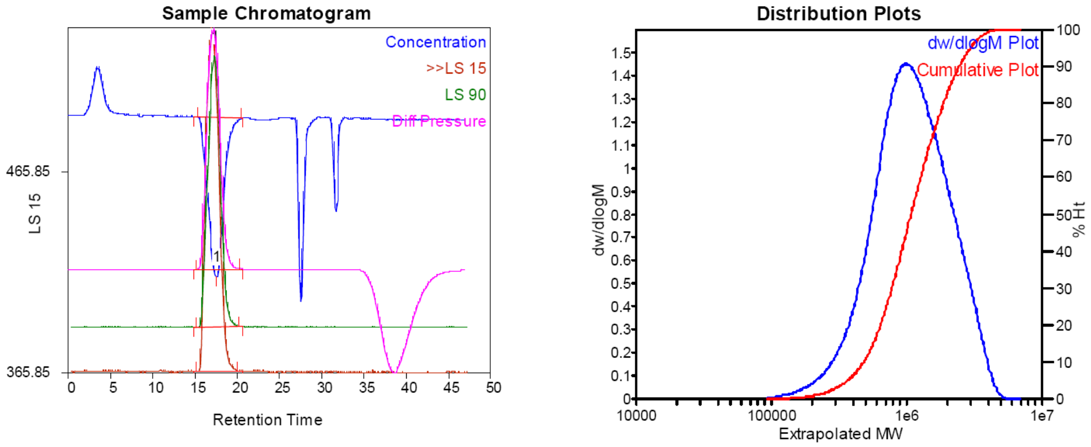

MW Averages

Peak No	Mp	Mn	Mw	Mz	Mz+1	Mv	PD
1	993565	860322	1284090	1784064	2288605	1195188	1.49257

Processed Peaks

Peak No	Name	Start RT (mins)	Max RT (mins)	End RT (mins)	Pk Height (mV)	% Height	Area (mV.secs)	% Area
1	Concentration	15.25	17.50	20.43	-23.1772	0	2737.09	100
2	LS 15	15.10	16.90	19.98	171.405	0	20399.4	100
3	LS 90	15.23	17.30	20.23	472.839	0	53865.7	100
4	Diff Pressure	15.22	17.18	20.32	630.773	0	72707	100

Supplementary Figure 34. GPC trace of the polymer from table 1, entry 2.

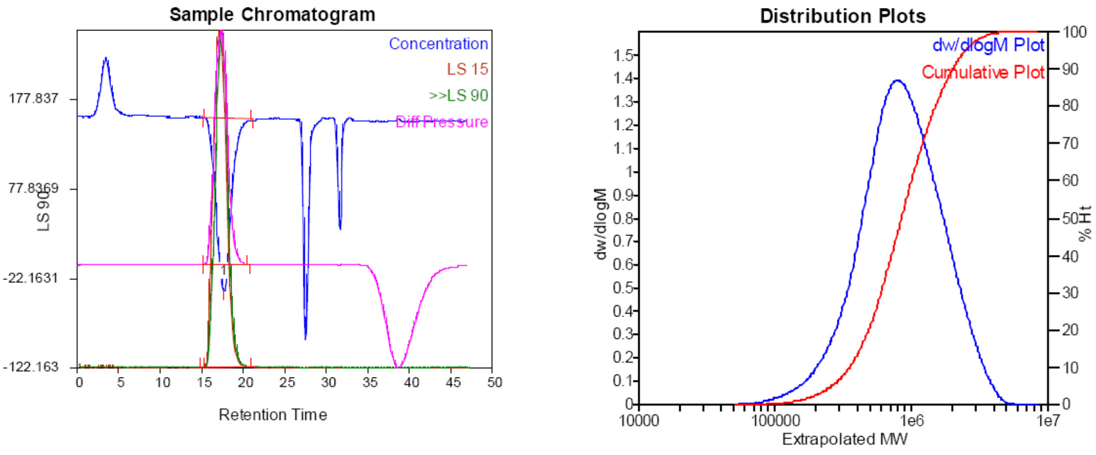

MW Averages

Peak No	Mp	Mn	Mw	Mz	Mz+1	Mv	PD
1	786512	647363	1033894	1522670	2065527	954179	1.59709

Processed Peaks

Peak No	Name	Start RT (mins)	Max RT (mins)	End RT (mins)	Pk Height (mV)	% Height	Area (mV.secs)	% Area
1	Concentration	15.33	17.70	20.93	-20.2744	0	2380.56	100
2	LS 15	14.92	17.15	20.92	121.702	0	14529.1	100
3	LS 90	15.25	17.45	20.87	376.675	0	42261.3	100
4	Diff Pressure	15.17	17.37	20.42	424.838	0	49179.5	100

Supplementary Figure 35. GPC trace of the polymer from table 1, entry 3.

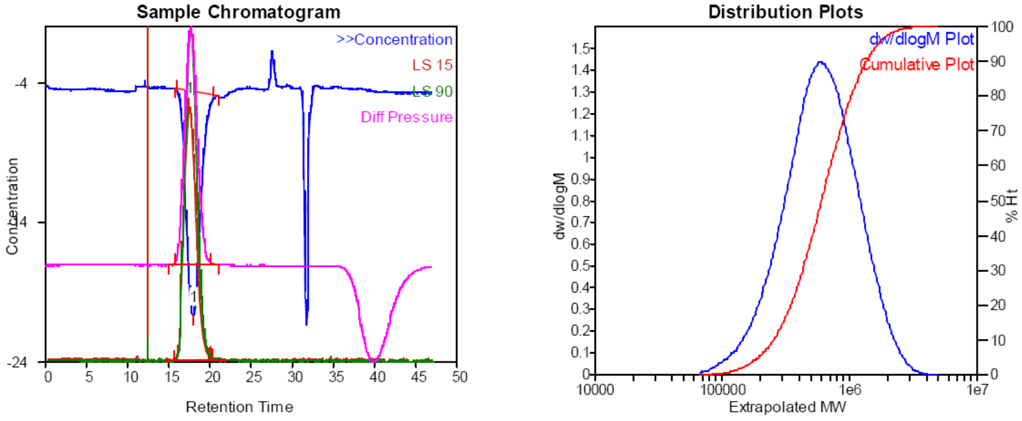

MW Averages

Peak No	Mp	Mn	Mw	Mz	Mz+1	Mv	PD
1	590800	470459	716915	1031711	1392268	675194	1.52386

Processed Peaks

Peak No	Name	Start RT (mins)	Max RT (mins)	End RT (mins)	Pk Height (mV)	% Height	Area (mV.secs)	% Area
1	Concentration	15.88	17.97	20.45	-16.0432	0	1763.92	100
2	LS 15	15.55	17.47	19.92	73.6107	0	7874.87	100
3	LS 90	15.67	17.63	20.27	258.234	0	26627.3	100
4	Diff Pressure	15.73	17.63	20.20	251.896	0	26845.8	100

Supplementary Figure 36. GPC trace of the polymer from table 1, entry 4.

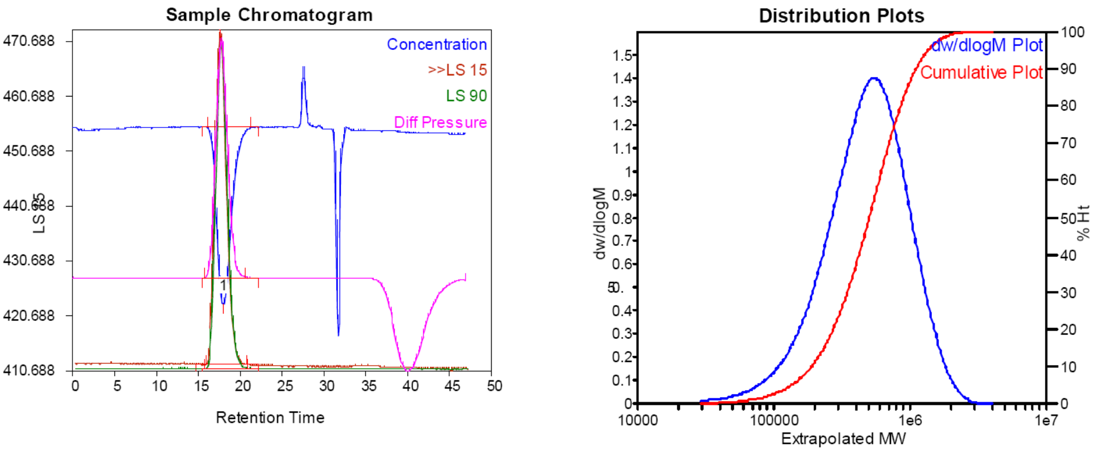

MW Averages

Peak No	Mp	Mn	Mw	Mz	Mz+1	Mv	PD
1	541930	356112	586185	842486	1113222	552884	1.64607

Processed Peaks

Peak No	Name	Start RT (mins)	Max RT (mins)	End RT (mins)	Pk Height (mV)	% Height	Area (mV.secs)	% Area
1	Concentration	16.02	17.97	21.23	-14.5779	0	1608	100
2	LS 15	15.87	17.57	20.73	60.9801	0	6026.53	100
3	LS 90	15.72	17.70	20.97	222.201	0	21500.4	100
4	Diff Pressure	15.80	17.70	20.55	207.833	0	20875.6	100

Supplementary Figure 37. GPC trace of the polymer from table 1, entry 5.

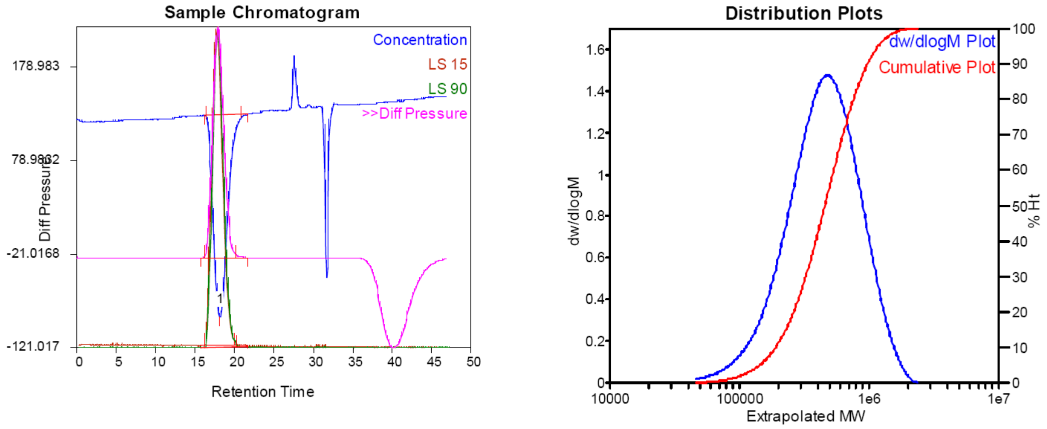

MW Averages

Peak No	Mp	Mn	Mw	Mz	Mz+1	Mv	PD
1	479553	350760	526227	724559	928458	500739	1.50025

Processed Peaks

Peak No	Name	Start RT (mins)	Max RT (mins)	End RT (mins)	Pk Height (mV)	% Height	Area (mV.secs)	% Area
1	Concentration	16.42	18.13	20.85	-18.8176	0	2060.71	100
2	LS 15	16.27	17.75	20.07	70.1476	0	6976.98	100
3	LS 90	16.30	17.83	20.28	263.917	0	25851.9	100
4	Diff Pressure	16.28	17.85	20.13	246.936	0	24848.3	100

Supplementary Figure 38. GPC trace of the polymer from table 1, entry 6.

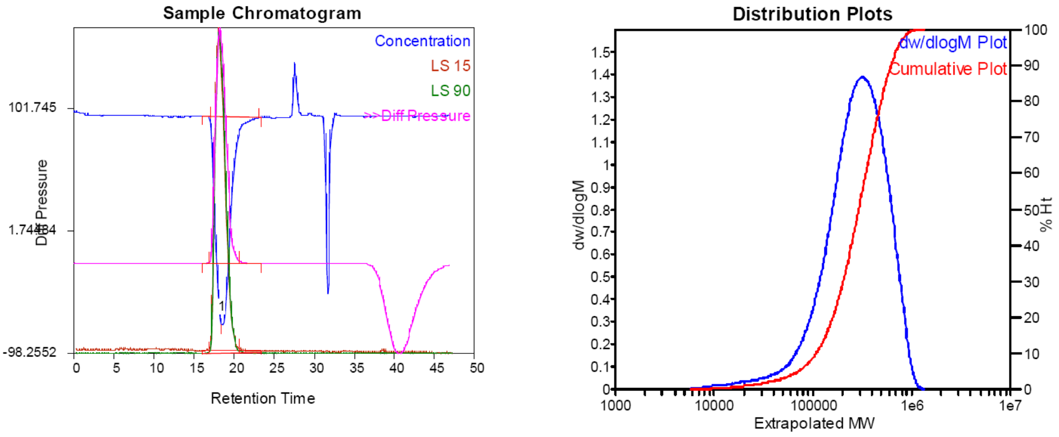

MW Averages

Peak No	Mp	Mn	Mw	Mz	Mz+1	Mv	PD
1	323632	178960	329998	451946	557278	313325	1.84397

Processed Peaks

Peak No	Name	Start RT (mins)	Max RT (mins)	End RT (mins)	Pk Height (mV)	% Height	Area (mV.secs)	% Area
1	Concentration	17.02	18.48	23.05	-19.4717	0	2206.03	100
2	LS 15	16.97	18.03	20.55	52.1402	0	4740.88	100
3	LS 90	16.88	18.10	20.67	211.51	0	19573.1	100
4	Diff Pressure	16.87	18.13	20.62	192.307	0	18491.1	100

Supplementary Figure 39. GPC trace of the polymer from table 1, entry 7.

DSC of Polymers

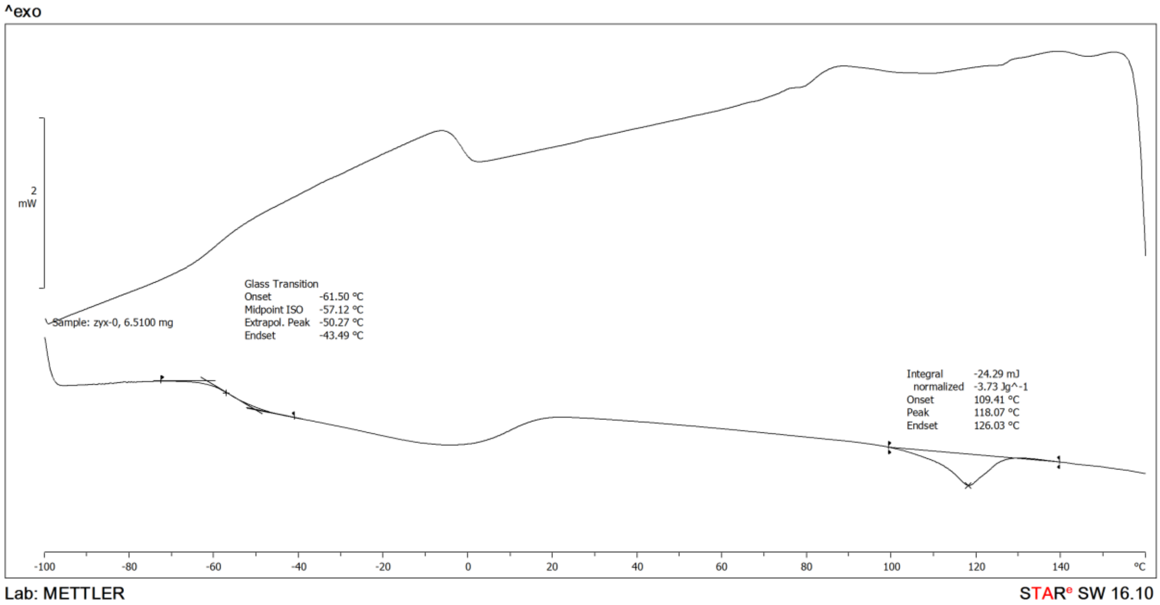

Supplementary Figure 40. DSC data of the polymer from table 2, entry 1.

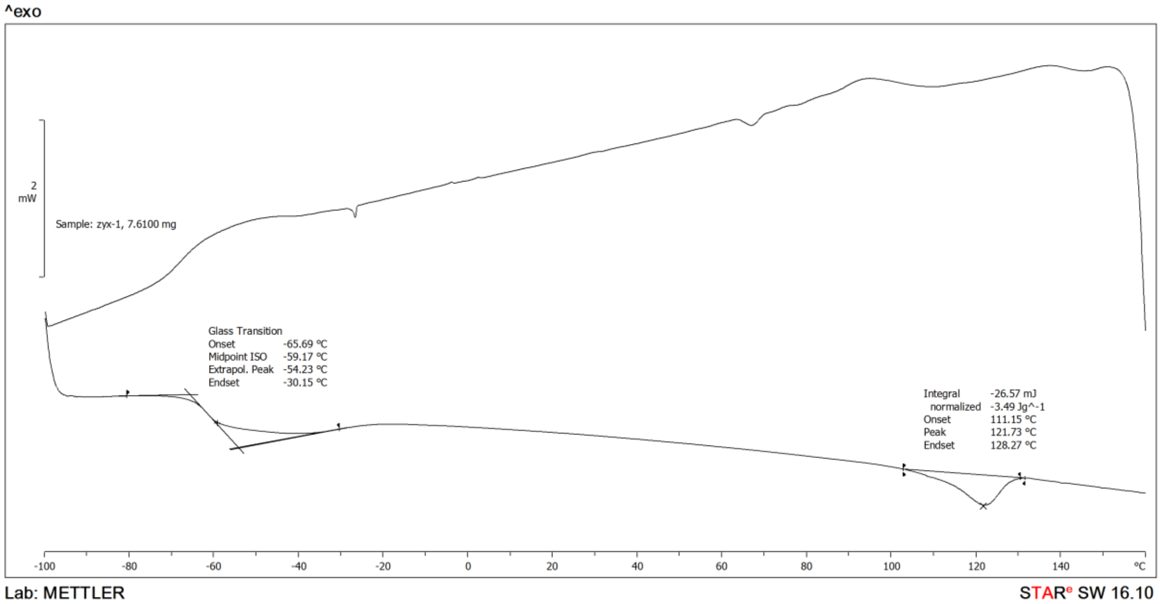

Supplementary Figure 41. DSC data of the polymer from table 2, entry 2.

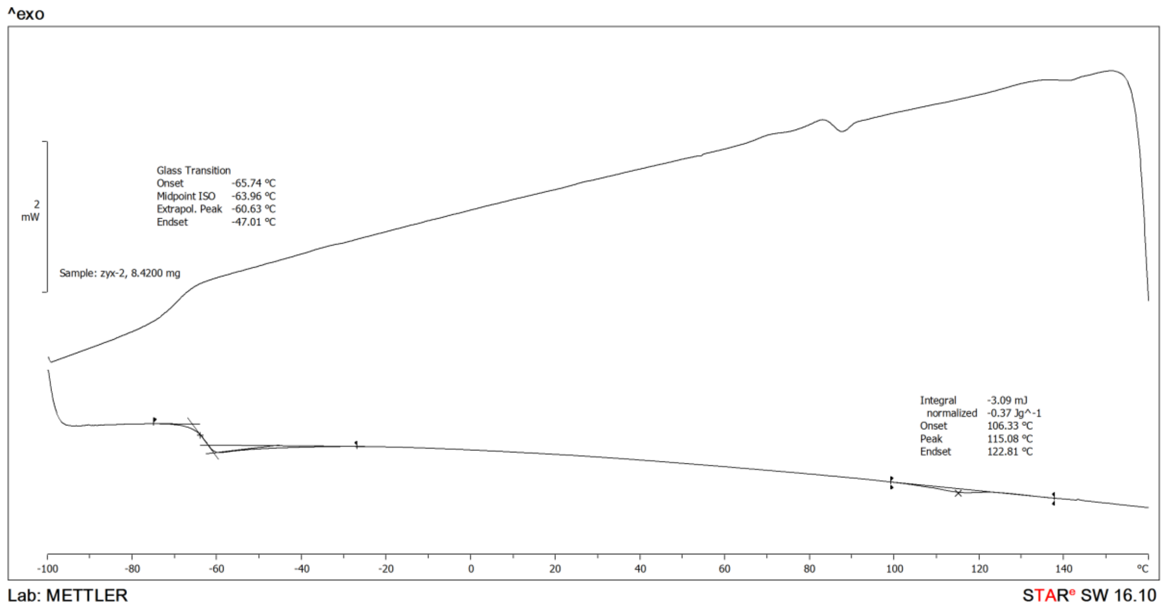

Supplementary Figure 42. DSC data of the polymer from table 2, entry 3.

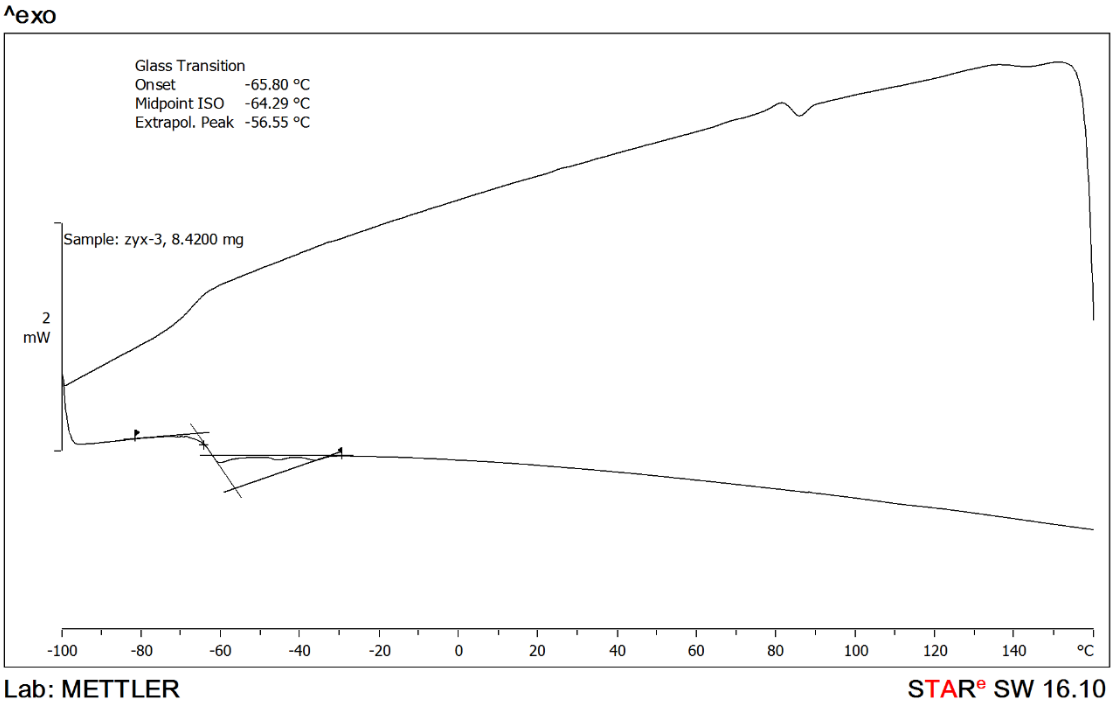

Supplementary Figure 43. DSC data of the polymer from table 2, entry 4.

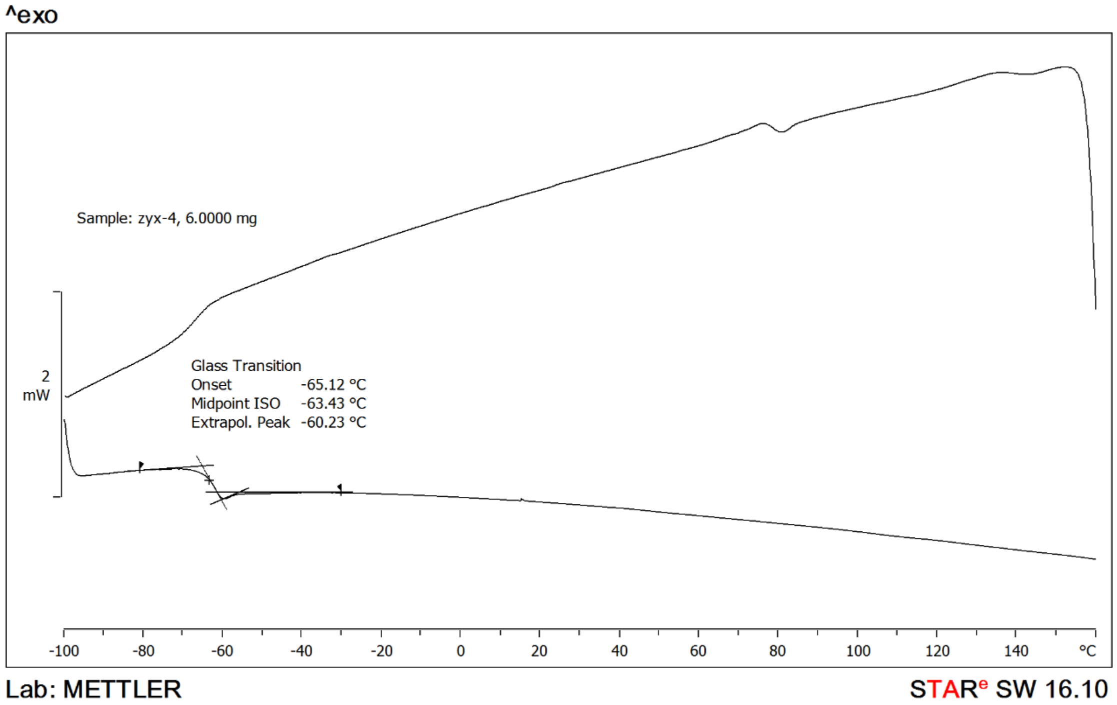

Supplementary Figure 44. DSC data of the polymer from table 2, entry 5.

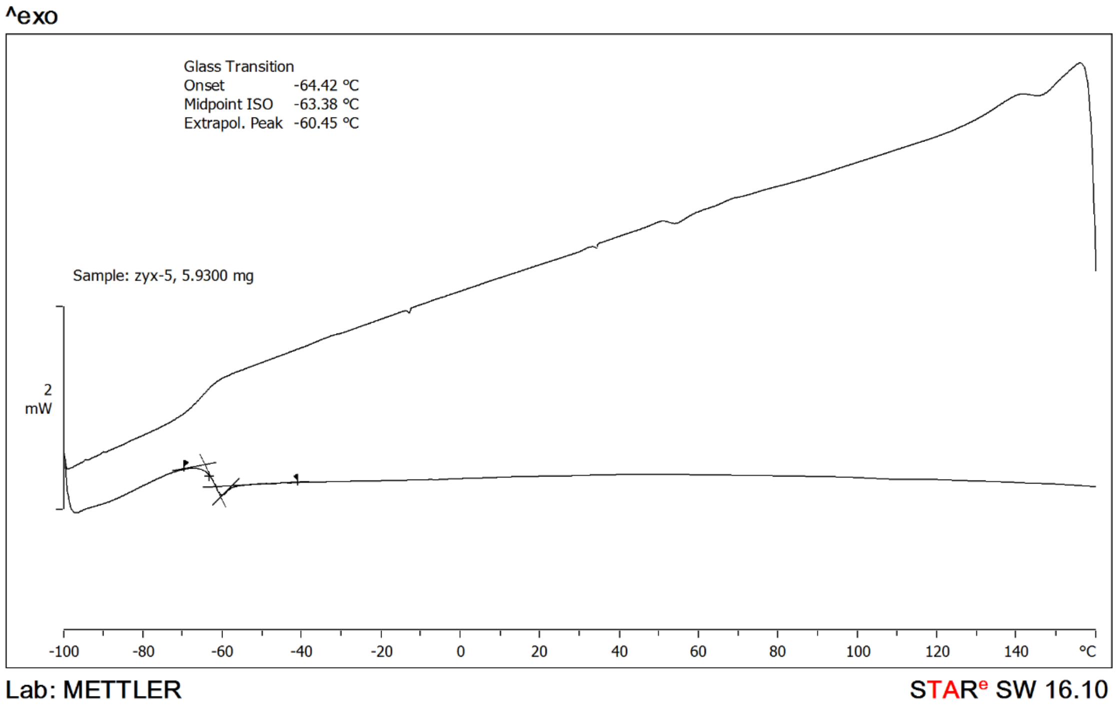

Supplementary Figure 45. DSC data of the polymer from table 2, entry 6.

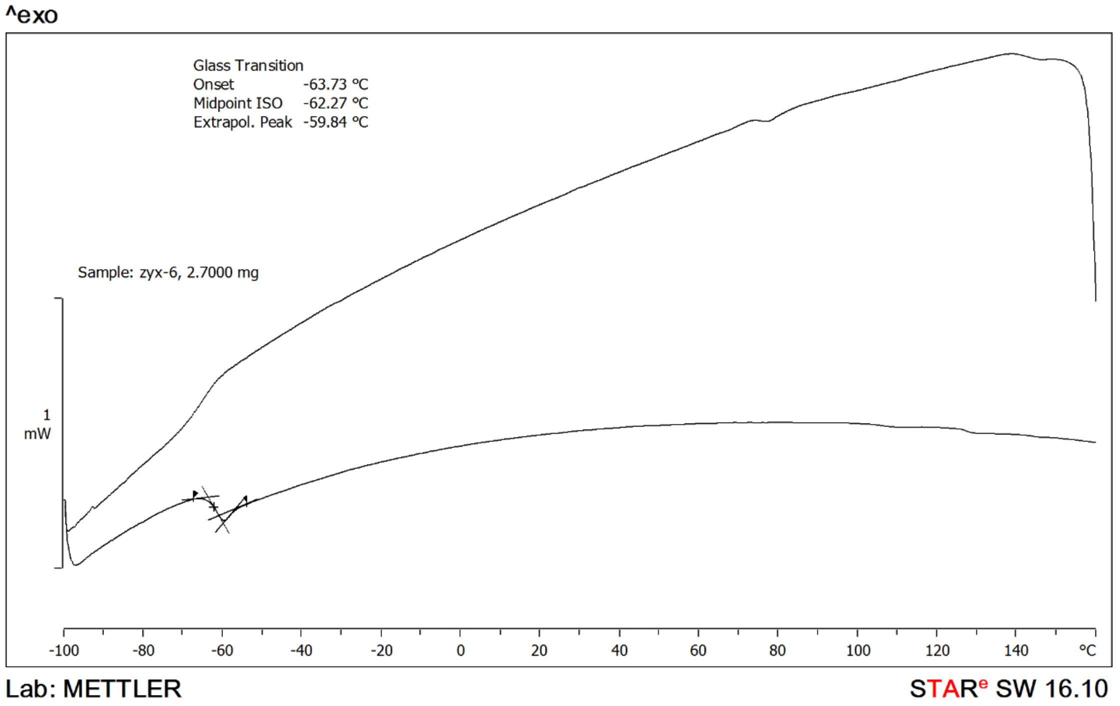

Supplementary Figure 46. DSC data of the polymer from table 2, entry 7.

Supplementary References

(1) Frisch, M. J.; Trucks, G. W.; Schlegel, H. B.; Scuseria, G. E.; Robb, M. A.; Cheeseman, J. R.; Scalmani, G.; Barone, V.; Mennucci, B.; Petersson, G. A.; Nakatsuji, H.; Caricato, M.; Li, X.; Hratchian, H. P.; Izmaylov, A. F.; Bloino, J.; Zheng, G.;. Sonnenberg, J. L; Hada, M.; Ehara, M.; Toyota, K.; Fukuda, R.; Hasegawa, J.; Ishida, M.; Nakajima, T.; Honda, Y.; Kitao, O.; Nakai, H.; Vreven, T.; Montgomery, J. A.; Peralta, J. E.; Ogliaro, F.; Bearpark, M.; Heyd, J. J.; Brothers, E.; Kudin, K. N.; Staroverov, V. N.; Kobayashi, R.; Normand, J.; Raghavachari, K.; Rendell, A.; Burant, J. C.; Iyengar, S. S.; Tomasi, J.; Cossi, M;. Rega, N.; Millam, J. M.; Klene, M.; Knox, J. E.; Cross, J. B.; Bakken, V.; Adamo, C.; Jaramillo, J.; Gomperts, R.; Stratmann, R. E.; Yazyev, O.; Austin, A. J.; Cammi, R.; Pomelli, C.; Ochterski, J. W. R.; Martin, L.; Morokuma, K.; Zakrzewski, V. G.; Voth, G. A.; Salvador, P.; Dannenberg, J. J.; Dapprich, S.; Daniels, A. D.; Farkas, Ö.; Foresman, J. B.; Ortiz, J. V.; Cioslowski, J.; Fox D. J. Gaussian 16 Revision C.01, Gaussian, Inc., Wallingford, CT, 2016.

(2) Perdew, J. P. Density-functional approximation for the correlation energy of the inhomogeneous electron gas. *Phys. Rev. B* **33**, 8822-8824 (1986).

(3) Perdew, J. P. Erratum: Density-functional approximation for the correlation energy of the inhomogeneous electron gas. *Phys. Rev. B* **34**, 7406-7406 (1986).

(4) Becke, A. D. Density-functional exchange-energy approximation with correct asymptotic behavior. *Phys. Rev. A* **38**, 3098-3100 (1988).

(5) Hay, P. J. Wadt, W. R., *J.* Ab initio effective core potentials for molecular calculations. Potentials for K to Au including the outermost core orbitals. *J. Chem. Phys.* **82**, 299 (1985).

(6) Grimme, S., Antony, J., Ehrlich, S., Krieg, H. A consistent and accurate ab initio parametrization of density functional dispersion correction (DFT-D) for the 94 elements H-Pu. *J. Chem. Phys.* **132**, 154104-15424 (2010).

(7) Grimme, S., Ehrlichm, S., Goerigk, L. Effect of the damping function in dispersion corrected density functional theory. *J. Comp. Chem.* **32**, 1456-1465 (2011).

(8) Marenich, A. V., Cramer, C. J., Truhlar, D. G. Universal solvation model based on solute electron density and on a continuum model of the solvent defined by the bulk dielectric constant and atomic surface tensions. *J. Phys. Chem. B* **113**, 6378-6396 (2009).

(9) Andrae, D., Häußermann, U., Dolg, M., Stoll, H., Preuß, H. Energy-adjustedab initio pseudopotentials for the second and third row transition elements. *Theor. Chim. Acta* **77**, 123-141 (1990).

(10) L. Falivene, T. Wiedemann, I. Göttker-Schnetmann, L. Caporaso, L. Cavallo, S. Mecking, Control of Chain Walking by Weak Neighboring Group Interactions in Unsymmetrical Catalysts. *J. Am. Chem. Soc.* **140**, 1305–1312 (2018).

(11) SMART, version 5.054; Bruker AXS Inc.: Madison, WI, 2000.

(12) SAINT and SADABS, version 6.22; Bruker AXS Inc.: Madison, WI, 2000.

(13) (a) G. M. Sheldrick, Acta Cryst. 2015, C71, 3-8. (b) O. V. Dolomanov, L. J. Bourhis, R. J. Gildea, J. A. K. Howard, H. Puschmann, OLEX2: A Complete Structure Solution, Refinement and Analysis Program. *J. Appl. Cryst.* **42**, 339-341 (2009).

(14) Spek, A. L. PLATON SQUEEZE: A Tool for the Calculation of the Disordered Solvent Contribution to the Calculated Structure Factors. *Acta Cryst.* **C71**, 9-18 (2015).
(15) Zhang, Y., Wang, C., Mecking, S. & Jian, Z. Ultrahigh Branching of Main-Chain-Functionalized Polyethylenes by Inverted Insertion Selectivity. *Angew. Chem. Int. Ed.* **59**, 14296-14302 (2020).
